# Supplementary material for: Effects of Energy Delivery Guided by Indirect Calorimetry in Critically Ill Patients: A Systematic Review and Meta-Analysis
Source: Nutrients. 2024 May 11;16(10):1452. doi: 10.3390/nu16101452 (PMC11124016; doi:10.3390/nu16101452)
Supplement: Supplementary file 1 [file nutrients-16-01452-s001.zip › nutrients-2977106-supplementary.pdf]

Supplemental file

Effects of Energy Delivery Guided by Indirect Calorimetry in Critically Ill Patients: A Systematic Review and Meta-Analysis

Shinichi Watanabe 1, Hiroo Izumino 2, Yudai Takatani 3, Rie Tsutsumi 4, Takahiro Suzuki 5, Hiroomi Tatsumi 6,
Ryo Yamamoto 7, Takeaki Sato 8, Tomoka Miyagi 9, Isao Miyajima 10, Kensuke Nakamura 11, Naoki Higashibeppu 12
and Joji Kotani 13,*
1 Department of Physical Therapy, Faculty of Rehabilitation, Gifu University of Health Science,
2-92 Higashiuzura, Gifu 500-8281, Japan; s-watanabe@gifuhoken.ac.jp
2 Acute and Critical Care Center, Nagasaki University Hospital, 1-7-1 Sakamoto, Nagasaki 852-8501, Japan; izumino@nagasaki-u.ac.jp
3 Department of Primary Care and Emergency Medicine, Kyoto University Hospital,
54 Shogoin-Kawahara-cho, Sakyo-ku, Kyoto 606-8507, Japan; takataniyu@kuhp.kyoto-u.ac.jp
4 Department of Nutrition and Metabolism, Institute of Biomedical Sciences, Tokushima University Graduate School, 3-18-15 Kuramoto, Tokushima 770-8503, Japan; rtsutsumi@tokushima-u.ac.jp
5 Department of Cardiovascular Medicine, St. Luke's International Hospital, 9-1 Akashi-cho, Chuo-ku,
Tokyo 104-8560, Japan; takasu.623@gmail.com
6 Department of Intensive Care Medicine, Sapporo Medical University School of Medicine, S1 W17, Chuo-ku,
Sapporo 060-8556, Japan; htatsumi@sapmed.ac.jp
7 Department of Emergency and Critical Care Medicine, Keio University School of Medicine,
35 Shinanomachi, Shinjuku-ku, Tokyo 160-8582, Japan; ryoyamamoto@keio.jp
8 Emergency Center, Tohoku University Hospital, 1-1 Seiryo-machi, Aoba-ku,

Sendai 980-8574, Japan; takesato@hkg.odn.ne.jp
9 Department of Nutrition, Yokosuka General Hospital, 2-36 Uwamachi, Yokosuka 238-8567, Japan; 10mtn.3@gmail.com
10 Department of Clinical Nutrition, Chikamori Hospital, 1-1-16 Okawasuzi, Kochi 780-8522, Japan; miyajimai@y6.dion.ne.jp
11 Department of Intensive Care, Yokohama City University Hospital, 3-9 Fukuura, Kanazawa-ku,
Yokohama 236-0064, Japan; mamashockpapashock@yahoo.co.jp
12 Department of Anesthesiology and Nutrition Support Team, Kobe City Medical Center General Hospital,
2-1-1 Minatojima Minamimachi, Chuo-ku, Kobe 650-0047, Japan; gashibe@hotmail.com
13 Division of Disaster and Emergency Medicine, Department of Surgery Related, Kobe University Graduate School of Medicine, 7-5-1 Kusunoki-cho, Chuo-ku, Kobe 650-0017, Japan
* Correspondence: kotanijo0412@gmail.com; Tel.: +81-78-382-6521

Contents

1. Reporting checklist.
2. Search strategies.
3. Studies excluded from full-text screening
4. Algorithm for the risk of bias judgment
5. Risk-of-bias summary and graph
6. Forest plot for outcomes.
7. Post-hoc analyses of primary outcomes
8. Subgroup analysis
9. Evidence profile

1. Reporting checklist.

Section and Topic	Item #	Checklist item	Location where item is reported
**TITLE**			
Title	1	Identify the report as a systematic review.	Page 1
**ABSTRACT**			
Abstract	2	See the PRISMA 2020 for Abstracts checklist.	Page 3
**INTRODUCTION**			
Rationale	3	Describe the rationale for the review in the context of existing knowledge.	Page 4
Objectives	4	Provide an explicit statement of the objective(s) or question(s) the review addresses.	Page 4
**METHODS**			
Eligibility criteria	5	Specify the inclusion and exclusion criteria for the review and how studies were grouped for the syntheses.	Page 4,5
Information sources	6	Specify all databases, registers, websites, organisations, reference lists and other sources searched or consulted to identify studies. Specify the date when each source was last searched or consulted.	Page 5
Search strategy	7	Present the full search strategies for all databases, registers and websites, including any filters and limits used.	Supplemental Table S2
Selection process	8	Specify the methods used to decide whether a study met the inclusion criteria of the review, including how many reviewers screened each record and each report retrieved, whether they worked independently, and if applicable, details of automation tools used in the process.	Page 5
Data collection process	9	Specify the methods used to collect data from reports, including how many reviewers collected data from each report, whether they worked independently, any processes for obtaining or confirming data from study investigators, and if applicable, details of automation tools used in the process.	Page 5,6
Data items	10a	List and define all outcomes for which data were sought. Specify whether all results that were compatible with each outcome domain in each study were sought (e.g. for all measures, time points, analyses), and if not, the methods used to decide which results to collect.	Page 6
	10b	List and define all other variables for which data were sought (e.g. participant and intervention characteristics, funding sources). Describe any assumptions made about any missing or unclear information.	Table 1
Study risk of	11	Specify the methods used to assess risk of bias in the included	Page 6

Section and Topic	Item #	Checklist item	Location where item is reported
bias assessment		studies, including details of the tool(s) used, how many reviewers assessed each study and whether they worked independently, and if applicable, details of automation tools used in the process.	
Effect measures	12	Specify for each outcome the effect measure(s) (e.g. risk ratio, mean difference) used in the synthesis or presentation of results.	Page 6,7
Synthesis methods	13a	Describe the processes used to decide which studies were eligible for each synthesis (e.g. tabulating the study intervention characteristics and comparing against the planned groups for each synthesis (item #5)).	Page 6, Table 1
	13b	Describe any methods required to prepare the data for presentation or synthesis, such as handling of missing summary statistics, or data conversions.	Page 5
	13c	Describe any methods used to tabulate or visually display results of individual studies and syntheses.	Page 5, Figure 1
	13d	Describe any methods used to synthesize results and provide a rationale for the choice(s). If meta-analysis was performed, describe the model(s), method(s) to identify the presence and extent of statistical heterogeneity, and software package(s) used.	Page 6
	13e	Describe any methods used to explore possible causes of heterogeneity among study results (e.g. subgroup analysis, meta-regression).	Page 6
	13f	Describe any sensitivity analyses conducted to assess robustness of the synthesized results.	Page 6
Reporting bias assessment	14	Describe any methods used to assess risk of bias due to missing results in a synthesis (arising from reporting biases).	Page 6,7
Certainty assessment	15	Describe any methods used to assess certainty (or confidence) in the body of evidence for an outcome.	Page 6,7
**RESULTS**			
Study selection	16a	Describe the results of the search and selection process, from the number of records identified in the search to the number of studies included in the review, ideally using a flow diagram.	Figure 1
	16b	Cite studies that might appear to meet the inclusion criteria, but which were excluded, and explain why they were excluded.	Supplemental Table S3
Study	17	Cite each included study and present its characteristics.	Table 1

Section and Topic	Item #	Checklist item	Location where item is reported
characteristics			
Risk of bias in studies	18	Present assessments of risk of bias for each included study.	Table 2
Results of individual studies	19	For all outcomes, present, for each study: (a) summary statistics for each group (where appropriate) and (b) an effect estimate and its precision (e.g. confidence/credible interval), ideally using structured tables or plots.	Figure 2
Results of syntheses	20a	For each synthesis, briefly summarise the characteristics and risk of bias among contributing studies.	Page 8,9
	20b	Present results of all statistical syntheses conducted. If meta-analysis was done, present for each the summary estimate and its precision (e.g. confidence/credible interval) and measures of statistical heterogeneity. If comparing groups, describe the direction of the effect.	Page 8,9, Figure 2
	20c	Present results of all investigations of possible causes of heterogeneity among study results.	Page 8,9, Supplemental Figure S2
	20d	Present results of all sensitivity analyses conducted to assess the robustness of the synthesized results.	Page 10, Figure 3
Reporting biases	21	Present assessments of risk of bias due to missing results (arising from reporting biases) for each synthesis assessed.	Page 8,9,10
Certainty of evidence	22	Present assessments of certainty (or confidence) in the body of evidence for each outcome assessed.	Page 8,9,10, Figure 2
**DISCUSSION**			
Discussion	23a	Provide a general interpretation of the results in the context of other evidence.	Page 11,12
	23b	Discuss any limitations of the evidence included in the review.	Page 12
	23c	Discuss any limitations of the review processes used.	Page 12,13
	23d	Discuss implications of the results for practice, policy, and future research.	Page 12,13
**OTHER INFORMATION**			
Registration and protocol	24a	Provide registration information for the review, including register name and registration number, or state that the review was not registered.	Page 14
	24b	Indicate where the review protocol can be accessed, or state that a protocol was not prepared.	Page 14
	24c	Describe and explain any amendments to information	Not

Section and Topic	Item #	Checklist item	Location where item is reported
		provided at registration or in the protocol.	applicable
Support	25	Describe sources of financial or non-financial support for the review, and the role of the funders or sponsors in the review.	Page 14
Competing interests	26	Declare any competing interests of review authors.	Page 14
Availability of data, code and other materials	27	Report which of the following are publicly available and where they can be found: template data collection forms; data extracted from included studies; data used for all analyses; analytic code; any other materials used in the review.	Page 15

Supplementary Table S1. Preferred Reporting Items for Systematic Review and Meta-Analysis (PRISMA) 2020 Checklist (From: Page MJ, McKenzie JE, Bossuyt PM, Boutron I, Hoffmann TC, Mulrow CD, et al. The PRISMA 2020 statement: an updated guideline for reporting systematic reviews. BMJ. 2021;372:n71. https://doi.org/10.1136/bmj.n71)

2. Search strategies

MEDLINE via PubMed search strategy (Searched in April 20, 2023)

#1	"Critical Illness"[MeSH Terms] OR "critical care"[MeSH Terms] OR "critical*"[Title/Abstract] OR "severe"[Title/Abstract] OR "intensive care units"[MeSH Terms] OR "intensive"[Title/Abstract] OR "ICU"[Title/Abstract] OR "CCU"[Title/Abstract] OR "coronary care unit*"[Title/Abstract] OR "HDU"[Title/Abstract] OR "RCU"[Title/Abstract] OR "respiratory care unit*"[Title/Abstract] OR "ITU"[Title/Abstract] OR "burn unit*"[Title/Abstract] OR "burn center*"[Title/Abstract] OR "recovery room*"[Title/Abstract] OR "respiration, artificial"[MeSH Terms] OR "Artificial Respiration"[Title/Abstract:~3] OR "Artificial Respirations"[Title/Abstract:~3] OR "Ventilation Mechanical"[Title/Abstract:~3] OR "Ventilations Mechanical"[Title/Abstract:~3] OR "shock"[MeSH Terms] OR "circulatory failure"[Title/Abstract:~3] OR "circulatory collapse"[Title/Abstract:~3] OR "multiple organ"[Title/Abstract] OR "MODS"[Title/Abstract] OR "Respiratory Insufficiency"[MeSH Terms] OR "respiratory failure"[Title/Abstract:~3] OR "respiratory failures"[Title/Abstract:~3] OR "respiratory depression"[Title/Abstract:~3] OR "ventilatory depression"[Title/Abstract:~3] OR "sepsis"[MeSH Terms] OR "sepsis"[Title/Abstract] OR "septic"[Title/Abstract] OR "Wounds and Injuries"[mesh] OR burn*[tiab] OR trauma*[tiab] OR Injur*[tiab] OR wound*[tiab]	4,537,953
#2	"calorimetry, indirect"[MeSH Terms] OR "Indirect Calorimetry"[Title/Abstract:~3] OR "indirect calorimet*"[Title/Abstract] OR "Calorimetry Respiration"[Title/Abstract:~3] OR "calorimetry respiration*"[Title/Abstract] OR "metabolic chamber"[Title/Abstract] OR "indirect energy measurement"[Title/Abstract:~3] OR "indirect calorie measurement"[Title/Abstract:~3]	9,078
#3	"randomized controlled trial"[Publication Type] OR "controlled clinical trial"[Publication Type] OR "randomized"[Title/Abstract] OR "placebo"[Title/Abstract] OR "drug therapy"[MeSH Subheading] OR "randomly"[Title/Abstract] OR "trial"[Title/Abstract] OR "groups"[Title/Abstract]) NOT ("animals"[MeSH Terms] NOT "humans"[MeSH Terms]	4,988,719

#4	#1 and #2 and #3	421
#5	"1940/01/01"[Date - Create] : "2023/03/31"[Date - Create]) and #4	421

The Cochrane Central Register of Controlled Trials (CENTRAL)

(Searched in April 20, 2023)

#1	[mh "Critical Illness"] or [mh "critical care"] or [mh "intensive care units"] or [mh "respiration, artificial"] or [mh shock] or [mh "respiratory insufficiency"] or [mh sepsis] or [mh "Wounds and Injuries"]	58781
#2	(critical*):ti,ab,kw or (severe):ti,ab,kw or (ICU):ti,ab,kw or (CCU):ti,ab,kw or (HDU):ti,ab,kw or (ITU):ti,ab,kw or (burn near/3 (unit* or center*)):ti,ab,kw or (recovery room*):ti,ab,kw or (artificial near/3 respirat*):ti,ab,kw or (ventilat* near/3 mechanical):ti,ab,kw or (circulat* near/3 (failure* or collapse*)):ti,ab,kw or ("multiple organ"):ti,ab,kw or (MODS):ti,ab,kw or (respirat* near/3 (failure* or depression*)):ti,ab,kw or (ventilat* near/3 (failure* or depression*)):ti,ab,kw or (sepsis):ti,ab,kw or (septic):ti,ab,kw or ("coronary care" unit*):ti,ab,kw or (RCU):ti,ab,kw or ("respiratory care" unit*):ti,ab,kw (burn*):ti,ab,kw or (trauma*):ti,ab,kw or (Injur*):ti,ab,kw or (wound*):ti,ab,kw (Word variations have been searched)	428503
#3	(intensive):ti,ab,kw	50902
#4	{OR #1-#3}	466397
#5	[mh "calorimetry, indirect"]	522
#6	(indirect* near/3 calorimetr*):ti,ab,kw or (calorimetr* near/3 respirat*):ti,ab,kw or (metabolic chamber):ti,ab,kw or (indirect near/2 energy near/2 measurement):ti,ab,kw or (indirect near/2 calorie near/2 measurement):ti,ab,kw (Word variations have been searched)	2742
#7	{OR #5-#6}	2742
#8	#4 and #7 in Trials	532

Igaku-Chuo-Zasshi (ICHUSHI) (Searched in April 20, 2023)

#1	(危篤/TH) or (末期状態/TA) or (救命/TA) or (集中治療/TA) or (クリティカル/TA) or (重症/TA) or (ICU/TH) or (集中治療/TA) or (ICU/TA) or (CCU/TA) or (冠疾患集中治療/TA) or (HDU/TA) or (RCU/TH) or (呼吸管理室/TA) or (呼吸集中治療/TA) or (ITU/TA) or (熱傷センター/TA) or (回復室/TA) or (リカバリー/TA) or (人工呼吸/TH) or (レスピレーター/TA) or (機械的換気/TA) or (人工換気/TA) or (人工呼吸/TA) or (ショック/TH) or (多臓器不全/TA) or (急性循環不全/TA) or (ショック/TA) or (循環虚脱/TA) or (呼吸不全/TH) or (呼吸不全/TA) or (呼吸機能不全/TA) or (敗血症/TH) or (敗血症/TA) or (敗血性/TA) or (創傷と損傷/TH) or (火傷/TA) or (熱傷/TA) or (外傷/TA) or (損傷/TA) or (けが/TA) or (怪我/TA) or (傷害/TA) or (創傷/TA) or (負傷/TA)	1,234,395
#2	(((間接的熱量測定/TH) or (間接/TA and 熱量/TA) or (間接/TA and カロリー/TA)))	1,054
#3	#1 or #2	336

Supplementary Table S2. Search strategies

3. Studies excluded from full-text screening

First investigator	Journal	Title	Reason for exclusion
Anbar, R	Clin Nutr Supplements	Tight calorie control in geriatric hip fracture patients: preliminary results of geriatric ticacos study	Congress abstract Protocol without results
Anbar, R	Clin Nutr Supplements	Tight calorie control (TICACOS) in geriatric hip fracture patients	Congress abstract
Azevedo, JRA	Clin Nutr Supplements	Optimized caloric-protein nutrition in critically ill patients. Impact on short and long-term outcomes	Congress abstract
Berger, MM	Clin Nutr Supplements	Supplemental parenteral nutrition does not alter substrate metabolism but improves immunity: the SPN2 randomized trial	Congress abstract
Berger, MM	Clin Nutr	Supplemental parenteral nutrition improves immunity with unchanged carbohydrate and protein metabolism in critically ill patients: The SPN2 randomized tracer study	Wrong intervention
Das, KC	ANESTHESIA	Nutrition in stroke patients: comparison of indirect calorimetry vs. Standard weight-based regimen	Congress abstract
Azevedo, JRA	BMC Anesthesiol	High-protein intake and early exercise in adult intensive care patients: a prospective, randomized controlled trial to evaluate the impact on functional outcomes	Wrong intervention
Dube, S	Journal of neurosurgical anesthesiology	Use of indirect calorimetry to assess outcome in stroke patients: a comparison with standard weight-based formula	Congress abstract
Leiderman, I	Clin Nutr Supplements	Implementation of nutritional support guidelines decreased days of mechanical ventilation and loss in surgical ICU	Congress abstract
(Not	https://www.medifin	Utilization of indirect	Protocol

applicable)	d.com/articles/clinical-trial/7164405	calorimetry for calculation of nutritional goals and its effect in ventilator-free days and muscle thickness in septic mechanically ventilated patients	without results
(Not applicable)	https://ichgcp.net/clinical-trials-registry/NCT02731144	Optimized Caloric-protein Nutrition in Septic and Septic Shock Patients	Protocol without results
(Not applicable)	https://classic.clinicaltrials.gov/ct2/show/NCT03249051	Optimization of Nutritional Therapy in Mechanically Ventilated, Critically Ill Patients	Protocol without results
Azevedo, JRA	Clin Nutr Supplements	Optimized Caloric-protein Nutrition in Critically Ill Patients	Congress abstract
(Not applicable)	https://classic.clinicaltrials.gov/ct2/show/NCT03440593	Indirect Calorimetry Usage and Effect in Ventilator-free Days and Muscle Thickness in Septic Ventilated Patients	Protocol without results
(Not applicable)	https://trialbulletin.com/lib/entry/ct-03871894	Indirect Calorimeter Based Study in Patients With Liver Cirrhosis	Protocol without results
(Not applicable)	https://classic.clinicaltrials.gov/ct2/show/NCT04479254	The Impact of IC-Guided Feeding Protocol on Clinical Outcomes in Critically Ill Patients (The IC-Study)	Protocol without results
(Not applicable)	http://www.who.int/trialsearch/Trial2.aspx?TrialID=ACTRN12615000205538	Determining energy expenditure during critical illness: a comparison of three instruments for indirect calorimetry in mechanically ventilated patients	Protocol without results
Japur, CC	J Crit Care	Harris-Benedict equation for critically ill patients: are there differences with indirect calorimetry?	Wrong study design
Zhi-Yong, R	Asia Pac J Clin Nutr	Comparison between measured and predicted resting energy	Wrong population
Shi, J	Zhonghua Wei Zhong Bing Ji Jiu Yi Xue	Application value of resting energy monitoring in nutritional support therapy for mechanical ventilation patients	Foreign language
Yang, X	Chin J Clin Nutri	Comparison of respiratory indirect calorimetry and	Foreign language

		Harris-Benedict coefficient in guiding energy target in patients with sepsis	

Supplementary Table S3. Studies excluded from full-text screening

4. Algorithm for the risk of bias judgment

Short-term mortality

	1.1	1.2	1.3	Risk
Jeffrey 1990	Y/PY	Y/PY	N/PN/NI	Low
Singer 2011	Y/PY	Y/PY	N/PN/NI	Low
Anbar 2014	Y/PY	Y/PY	N/PN/NI	Low
Allingstrup 2017	Y/PY	Y/PY	N/PN/NI	Low
Gonzalez-Granda 2019	Y/PY	Y/PY	N/PN/NI	Low
Azevedo 2019	Y/PY	Y/PY	N/PN/NI	Low
Singer 2020	Y/PY	Y/PY	N/PN/NI	Low

	2.1	2.2	2.3	2.4	2.5	2.6	2.7	Risk
Jeffrey 1990	EY/PY/NI	EY/PY/NI	N/PN			Y/PY		Low
Singer 2011	EY/PY/NI	EY/PY/NI	N/PN			Y/PY		Low
Anber 2014	EY/PY/NI	EY/PY/NI	Y/PY	N/PN		Y/PY		Some concerns
Allingstrup 2017	EY/PY/NI	EY/PY/NI	N/PN			Y/PY		Low
Gonzalez-Granda 2019	EY/PY/NI	EY/PY/NI	N/PN			Y/PY		Low
Azevedo 2019	EY/PY/NI	EY/PY/NI	Y/PY	N/PN		Y/PY		Some concerns
Singer 2020	EY/PY/NI	EY/PY/NI	N/PN			Y/PY		Low

	3.1	3.2	3.3	3.4	Risk
Jeffrey 1990	N/PN/NI	N/PN	Y/PY/NI	N/PN	Some concerns
Singer 2011	N/PN/NI	N/PN	N/PN		Low
Anbar 2014	N/PN/NI	N/PN	N/PN		Low

Allingstrup 2017	N/PN/NI	N/PN	N/PN		Low	
Gonzalez-Granda 2019	N/PN/NI	N/PN	N/PN		Low	
Azevedo 2019	N/PN/NI	N/PN	Y/PY/NI	N/PN	Some concerns	
Singer 2020	N/PN/NI	N/PN	N/PN		Low	
	4.1	4.2	4.3	4.4	4.5	Risk
Jeffrey 1990	N/PN/NI	N/PN	N/PN			Low
Singer 2011	N/PN/NI	N/PN	N/PN			Low
Anbar 2014	N/PN/NI	N/PN	N/PN			Low
Allingstrup 2017	N/PN/NI	N/PN	N/PN			Low
Gonzalez-Granda 2019	N/PN/NI	N/PN	N/PN			Low
Azevedo 2019	N/PN/NI	N/PN	Y/PY/NI	Y/PY/NI	N/PN	Some concerns
Singer 2020	N/PN/NI	N/PN	N/PN			Low
	5.1	5.2	5.3	Risk		
Jeffrey 1990	N/PN/NI	Both<br>N/PN	Both<br>N/PN	Some concerns		
Singer 2011	Y/PY	Both<br>N/PN	Both<br>N/PN	Low		
Anbar 2014	N/PN/NI	Both<br>N/PN	Both<br>N/PN	Some concerns		
Allingstrup 2017	Y/PY	Both<br>N/PN	Both<br>N/PN	Low		
Gonzalez-Granda 2019	Y/PY	Both<br>N/PN	Both<br>N/PN	Low		

Azevedo 2019	N/PN/NI	Both N/PN	Both N/PN	Some concerns
Singer 2020	Y/PY	Both N/PN	Both N/PN	Low

Length of ICU stay

	1.1	1.2	1.3	Risk
Jeffrey 1990	Y/PY	Y/PY	N/PN/NI	Low
Singer 2011	Y/PY	Y/PY	N/PN/NI	Low
Allingstrup 2017	Y/PY	Y/PY	N/PN/NI	Low
Azevedo 2019	Y/PY	Y/PY	N/PN/NI	Low
Gonzalez-Granda 2019	Y/PY	Y/PY	N/PN/NI	Low
Singer 2020	Y/PY	Y/PY	N/PN/NI	Low
Farah 2021	Y/PY	Y/PY	N/PN/NI	Low

	2.1	2.2	2.3	2.4	2.5	2.6	2.7	Risk
Jeffrey 1990	EY/PY/NI	EY/PY/NI	N/PN			Y/PY		Low
Singer 2011	EY/PY/NI	EY/PY/NI	N/PN			Y/PY		Low
Allingstrup 2017	EY/PY/NI	EY/PY/NI	N/PN			Y/PY		Low
Azevedo 2019	EY/PY/NI	EY/PY/NI	Y/PY	N/PN		Y/PY		Some concerns
Gonzalez-Granda 2019	EY/PY/NI	EY/PY/NI	N/PN			Y/PY		Low
Singer 2020	EY/PY/NI	EY/PY/NI	N/PN			Y/PY		Low
Farah 2021	EY/PY/NI	EY/PY/NI	N/PN			Y/PY		Low

	3.1	3.2	3.3	3.4	Risk
Jeffrey 1990	N/PN/NI	N/PN	Y/PY/NI	N/PN	Some concerns

Singer 2011	N/PN/NI	N/PN	Y/PY/NI	N/PN	Some concerns
Allingstrup 2017	N/PN/NI	N/PN	N/PN		Low
Azevedo 2019	N/PN/NI	N/PN	Y/PY/NI	N/PN	Some concerns
Gonzalez-Granda 2019	N/PN/NI	N/PN	Y/PY/NI	N/PN	Some concerns
Singer 2020	N/PN/NI	N/PN	N/PN		Low
Farah 2021	N/PN/NI	N/PN	N/PN		Low

	4.1	4.2	4.3	4.4	4.5	Risk
Jeffrey 1990	N/PN/NI	N/PN	Y/PY/NI	Y/PY/NI	N/PN	Some concerns
Singer 2011	N/PN/NI	N/PN	N/PN			Low
Allingstrup 2017	N/PN/NI	N/PN	N/PN			Low
Azevedo 2019	N/PN/NI	N/PN	Y/PY/NI	Y/PY/NI	N/PN	Some concerns
Gonzalez-Granda 2019	N/PN/NI	N/PN	N/PN			Low
Singer 2020	N/PN/NI	N/PN	Y/PY/NI	Y/PY/NI	N/PN	Some concerns
Farah 2021	N/PN/NI	N/PN	N/PN			Low

	5.1	5.2	5.3	Risk
Jeffrey 1990	N/PN/NI	Both<br>N/PN	Both<br>N/PN	Some concerns
Singer 2011	Y/PY	Both<br>N/PN	Both<br>N/PN	Low
Allingstrup 2017	Y/PY	Both<br>N/PN	Both<br>N/PN	Low
Azevedo 2019	N/PN/NI	Both<br>N/PN	Both<br>N/PN	Some concerns

Gonzalez-Granda 2019	N/PN/NI	Both N/PN	Both N/PN	Some concerns
Singer 2020	Y/PY	Both N/PN	Both N/PN	Low
Farah 2021	N/PN/NI	Both N/PN	Both N/PN	Some concerns

Duration of mechanical ventilation

	1.1	1.2	1.3	Risk
Singer 2011	Y/PY	Y/PY	N/PN/NI	Low
Landes 2016	Y/PY	Y/PY	N/PN/NI	Low
Allingstrup 2017	Y/PY	Y/PY	N/PN/NI	Low
Azevedo 2019	Y/PY	Y/PY	N/PN/NI	Low
Gonzalez-Granda 2019	Y/PY	Y/PY	N/PN/NI	Low
Singer 2020	Y/PY	Y/PY	N/PN/NI	Low
Farah 2021	Y/PY	Y/PY	N/PN/NI	Low

	2.1	2.2	2.3	2.4	2.5	2.6	2.7	Risk
Singer 2011	EY/PY/NI	EY/PY/NI	N/PN			Y/PY		Low
Landes 2016	EY/PY/NI	EY/PY/NI	N/PN			Y/PY		Low
Allingstrup 2017	EY/PY/NI	EY/PY/NI	N/PN			Y/PY		Low
Azevedo 2019	EY/PY/NI	EY/PY/NI	Y/PY	N/PN		Y/PY		Some concerns
Gonzalez-Granda 2019	EY/PY/NI	EY/PY/NI	N/PN			Y/PY		Low
Singer 2020	EY/PY/NI	EY/PY/NI	N/PN			Y/PY		Low
Farah 2021	EY/PY/NI	EY/PY/NI	N/PN			Y/PY		Low

	3.1	3.2	3.3	3.4	Risk
Singer 2011	N/PN/NI	N/PN	Y/PY/NI	N/PN	Some concerns
Landes 2016	N/PN/NI	N/PN	N/PN		Low
Allingstrup 2017	N/PN/NI	N/PN	N/PN		Low
Azevedo 2019	N/PN/NI	N/PN	Y/PY/NI	N/PN	Some concerns
Gonzalez-Granda 2019	N/PN/NI	N/PN	N/PN		Low
Singer 2020	N/PN/NI	N/PN	N/PN		Low
Farah 2021	N/PN/NI	N/PN	N/PN		Low

	4.1	4.2	4.3	4.4	4.5	Risk
Singer 2011	N/PN/NI	N/PN	N/PN			Low
Landes 2016	N/PN/NI	N/PN	N/PN			Low
Allingstrup 2017	N/PN/NI	N/PN	N/PN			Low
Azevedo 2019	N/PN/NI	N/PN	Y/PY/NI	Y/PY/NI	N/PN	Some concerns
Gonzalez-Granda 2019	N/PN/NI	N/PN	Y/PY/NI	Y/PY/NI	N/PN	Some concerns
Singer 2020	N/PN/NI	N/PN	Y/PY/NI	Y/PY/NI	N/PN	Some concerns
Farah 2021	N/PN/NI	N/PN	N/PN			Low

	5.1	5.2	5.3	Risk
Singer 2011	Y/PY	Both<br>N/PN	Both<br>N/PN	Low
Landes 2016	Y/PY	Both<br>N/PN	Both<br>N/PN	Low
Allingstrup 2017	Y/PY	Both<br>N/PN	Both<br>N/PN	Low

Azevedo 2019	N/PN/NI	Both<br>N/PN	Both<br>N/PN	Some concerns
Gonzalez-Granda 2019	Y/PY	Both<br>N/PN	Both<br>N/PN	Low
Singer 2020	Y/PY	Both<br>N/PN	Both<br>N/PN	Low
Farah 2021	N/PN/NI	Both<br>N/PN	Both<br>N/PN	Some concerns

All infections

	1.1	1.2	1.3	Risk
Singer 2011	Y/PY	Y/PY	N/PN/NI	Low
Anbar 2014	Y/PY	Y/PY	N/PN/NI	Low
Allingstrup 2017	Y/PY	Y/PY	N/PN/NI	Low
Singer 2020	Y/PY	Y/PY	N/PN/NI	Low

	2.1	2.2	2.3	2.4	2.5	2.6	2.7	Risk
Singer 2011	EY/PY/NI	EY/PY/NI	N/PN			Y/PY		Low
Anbar 2014	EY/PY/NI	EY/PY/NI	Y/PY	N/PN		Y/PY		Some concerns
Allingstrup 2017	EY/PY/NI	EY/PY/NI	N/PN			Y/PY		Low
Singer 2020	EY/PY/NI	EY/PY/NI	N/PN			Y/PY		Low

	3.1	3.2	3.3	3.4	Risk
Singer 2011	N/PN/NI	N/PN	Y/PY/NI	N/PN	Some concerns
Anber 2014	N/PN/NI	N/PN	N/PN		Low
Allingstrup 2017	N/PN/NI	N/PN	N/PN		Low

Singer 2020	N/PN/NI	N/PN	N/PN		Low			
	4.1	4.2	4.3	4.4	4.5	Risk		
Singer 2011	N/PN/NI	N/PN	N/PN			Low		
Anbar 2014	N/PN/NI	N/PN	N/PN			Low		
Allingstrup 2017	N/PN/NI	N/PN	N/PN			Low		
Singer 2020	N/PN/NI	N/PN	Y/PY/NI	Y/PY/NI	N/PN	Some concerns		
	5.1	5.2	5.3	Risk				
Singer 2011	Y/PY	Both<br>N/PN	Both<br>N/PN	Low				
Anbar 2014	N/PN/NI	Both<br>N/PN	Both<br>N/PN	Some concerns				
Allingstrup 2017	Y/PY	Both<br>N/PN	Both<br>N/PN	Low				
Singer 2020	Y/PY	Both<br>N/PN	Both<br>N/PN	Low				

Ventilator-associated pneumonia

	1.1	1.2	1.3	Risk				
Singer 2011	Y/PY	Y/PY	N/PN/NI	Low				
Anbar 2014	Y/PY	Y/PY	N/PN/NI	Low				
Allingstrup 2017	Y/PY	Y/PY	N/PN/NI	Low				
Singer 2020	Y/PY	Y/PY	N/PN/NI	Low				
	2.1	2.2	2.3	2.4	2.5	2.6	2.7	Risk
Singer 2011	EY/PY/NI	EY/PY/NI	N/PN			Y/PY		Low

Anbar 2014	EY/PY/NI	EY/PY/NI	Y/PY	N/PN		Y/PY	Some concerns
Allingstrup 2017	EY/PY/NI	EY/PY/NI	N/PN			Y/PY	Low
Singer 2020	EY/PY/NI	EY/PY/NI	N/PN			Y/PY	Low
	3.1	3.2	3.3	3.4	Risk		
Singer 2011	N/PN/NI	N/PN	Y/PY/NI	N/PN	Some concerns		
Anbar 2014	N/PN/NI	N/PN	N/PN		Low		
Allingstrup 2017	N/PN/NI	N/PN	N/PN		Low		
Singer 2020	N/PN/NI	N/PN	N/PN		Low		
	4.1	4.2	4.3	4.4	4.5	Risk	
Singer 2011	N/PN/NI	N/PN	N/PN			Low	
Anbar 2014	N/PN/NI	N/PN	N/PN			Low	
Allingstrup 2017	N/PN/NI	N/PN	N/PN			Low	
Singer 2020	N/PN/NI	N/PN	Y/PY/NI	Y/PY/NI	N/PN	Some concerns	
	5.1	5.2	5.3	Risk			
Singer 2011	Y/PY	Both N/PN	Both N/PN	Low			
Anbar 2014	N/PN/NI	Both N/PN	Both N/PN	Some concerns			
Allingstrup 2017	Y/PY	Both N/PN	Both N/PN	Low			
Singer 2020	Y/PY	Both N/PN	Both N/PN	Low			
**physical functions**							

(physical component summary)

	1.1	1.2	1.3	Risk
Azevedo 2019	Y/PY	Y/PY	N/PN/NI	Low
Allingstrup 2017	Y/PY	Y/PY	N/PN/NI	Low

	2.1	2.2	2.3	2.4	2.5	2.6	2.7	Risk
Azevedo 2019	EY/PY/NI	EY/PY/NI	Y/PY	N/PN		Y/PY		Some concerns
Allingstrup 2017	EY/PY/NI	EY/PY/NI	N/PN			Y/PY		Low

	3.1	3.2	3.3	3.4	Risk
Azevedo 2019	N/PN/NI	N/PN	Y/PY/NI	N/PN	Some concerns
Allingstrup 2017	N/PN/NI	N/PN	N/PN		Low

	4.1	4.2	4.3	4.4	4.5	Risk
Azevedo 2019	N/PN/NI	N/PN	Y/PY/NI	Y/PY/NI	N/PN	Some concerns
Allingstrup 2017	N/PN/NI	N/PN	N/PN			Low

	5.1	5.2	5.3	Risk
Azevedo 2019	N/PN/NI	Both N/PN	Both N/PN	Some concerns
Allingstrup 2017	Y/PY	Both N/PN	Both N/PN	Low

Adverse events (kidney)

	1.1	1.2	1.3	Risk

Singer 2011	Y/PY/NI	Y/PY	N/PN/NI	Low				
Singer 2020	Y/PY/NI	Y/PY	N/PN/NI	Low				
	2.1	2.2	2.3	2.4	2.5	2.6	2.7	Risk
Singer 2011	EY/PY/NI	EY/PY/NI	N/PN			Y/PY		Low
Singer 2020	EY/PY/NI	EY/PY/NI	N/PN			Y/PY		Low
	3.1	3.2	3.3	3.4	Risk			
Singer 2011	N/PN/NI	N/PN	Y/PY/NI	N/PN	Some concerns			
Singer 2020	N/PN/NI	N/PN	N/PN		Low			
	4.1	4.2	4.3	4.4	4.5	Risk		
Singer 2011	N/PN/NI	N/PN	N/PN			Low		
Singer 2020	N/PN/NI	N/PN	Y/PY/NI	Y/PY/NI	N/PN	Some concerns		
	5.1	5.2	5.3	Risk				
Singer 2011	Y/PY	Both N/PN	Both N/PN	Low				
Singer 2020	Y/PY	Both N/PN	Both N/PN	Low				

Adverse events (liver)

	1.1	1.2	1.3	Risk				
Singer 2011	Y/PY/NI	Y/PY	N/PN/NI	Low				
Singer 2020	Y/PY/NI	Y/PY	N/PN/NI	Low				
	2.1	2.2	2.3	2.4	2.5	2.6	2.7	Risk
Singer 2011	EY/PY/NI	EY/PY/NI	N/PN			Y/PY		Low
Singer 2020	EY/PY/NI	EY/PY/NI	N/PN			Y/PY		Low

	3.1	3.2	3.3	3.4	Risk	
Singer 2011	N/PN/NI	N/PN	Y/PY/NI	N/PN	Some concerns	
Singer 2020	N/PN/NI	N/PN	N/PN		Low	
	4.1	4.2	4.3	4.4	4.5	Risk
Singer 2011	N/PN/NI	N/PN	N/PN			Low
Singer 2020	N/PN/NI	N/PN	Y/PY/NI	Y/PY/NI	N/PN	Some concerns
	5.1	5.2	5.3	Risk		
Singer 2011	Y/PY	Both<br>N/PN	Both<br>N/PN	Low		
Singer 2020	Y/PY	Both<br>N/PN	Both<br>N/PN	Low		

Supplementary Table S4. Algorithm for the risk of bias judgment

5. Risk-of-bias summary and graph

A. Short-term mortality

A-1. Risk-of-bias summary: Review authors' judgements about each risk of bias item for each included study.

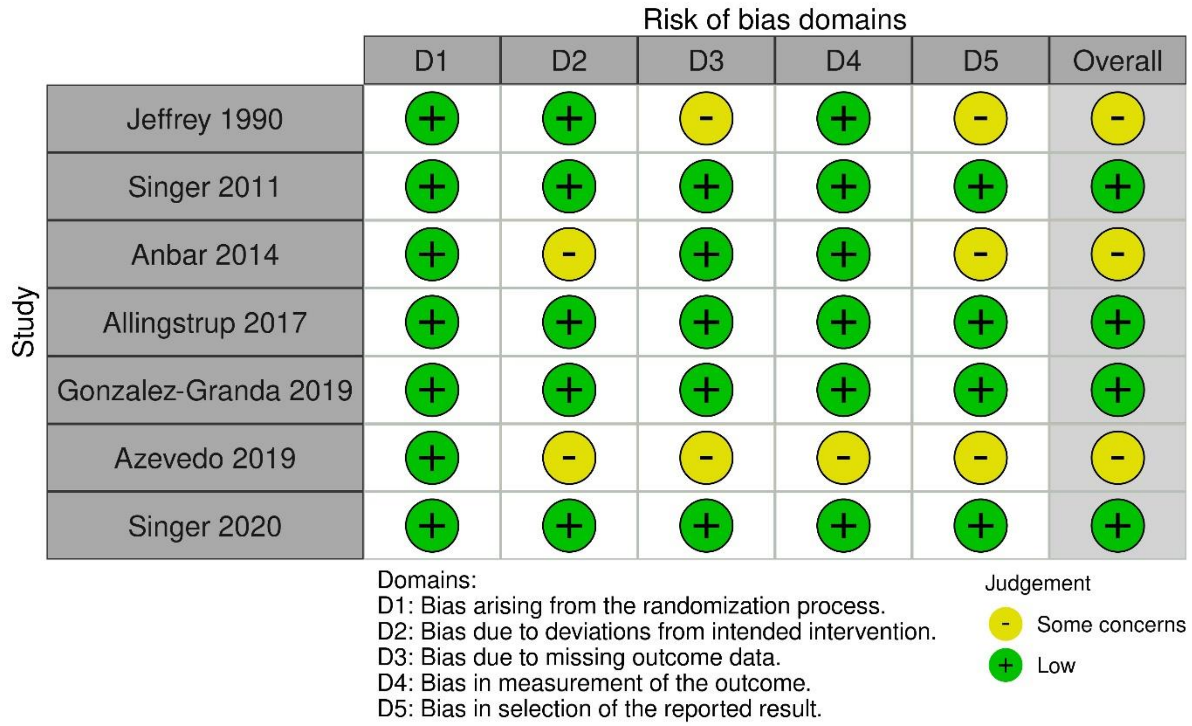

A-2. Risk-of-bias graph: Review authors' judgements about each risk of bias item presented as percentages across all included studies.

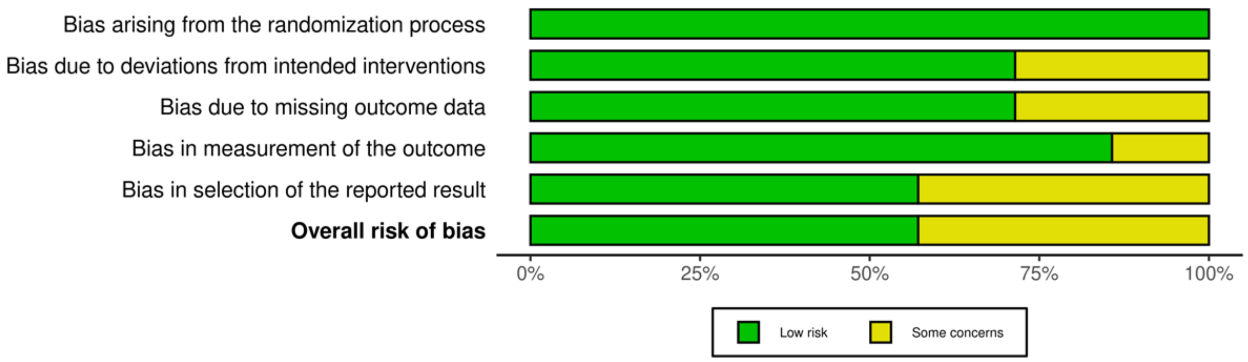

A-3. Forest plot of comparison: Mortality

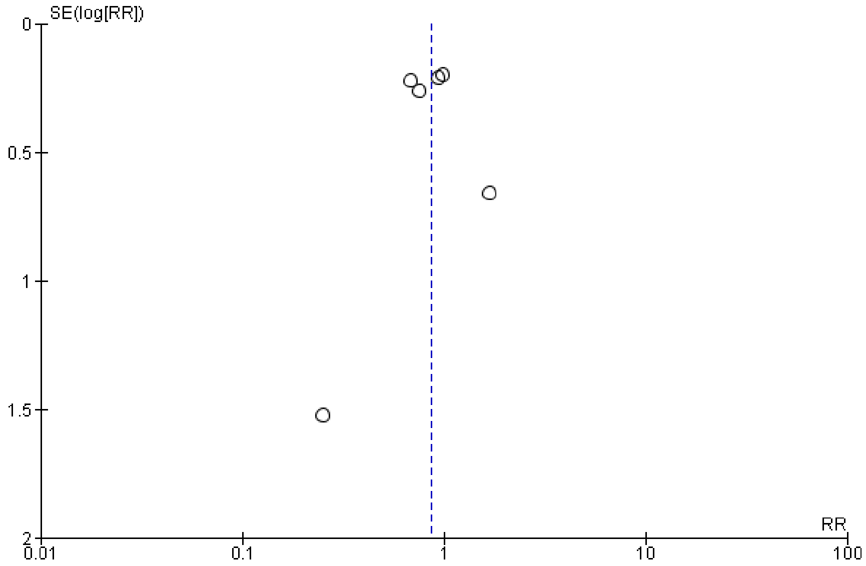

B. Length of ICU stay

B-1. Risk-of-bias summary: Review authors' judgements about each risk of bias item for each included study.

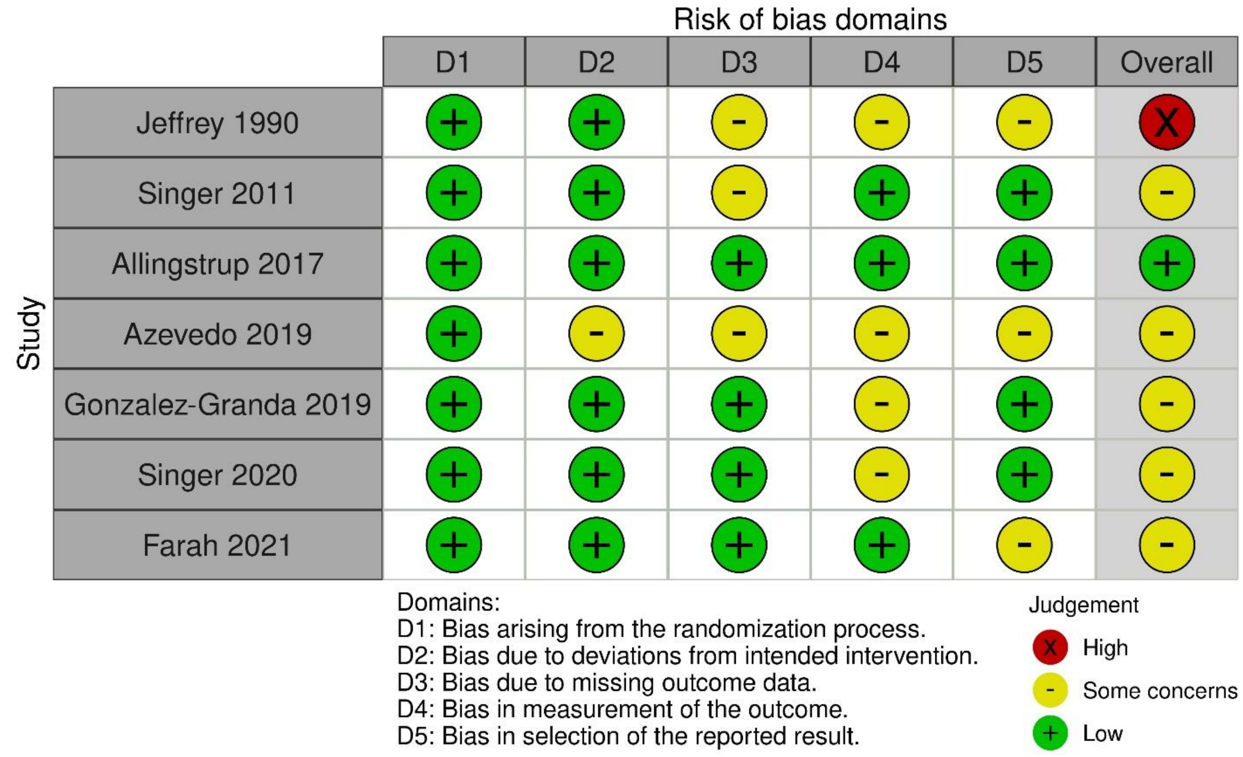

B-2. Risk-of-bias graph: Review authors' judgements about each risk of bias item presented as percentages across all included studies.

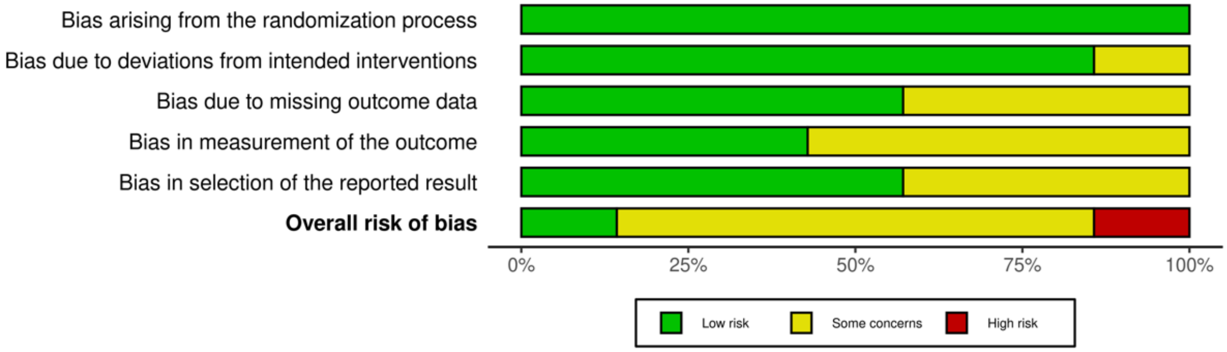

B-3. Forest plot of comparison: Length of ICU stay

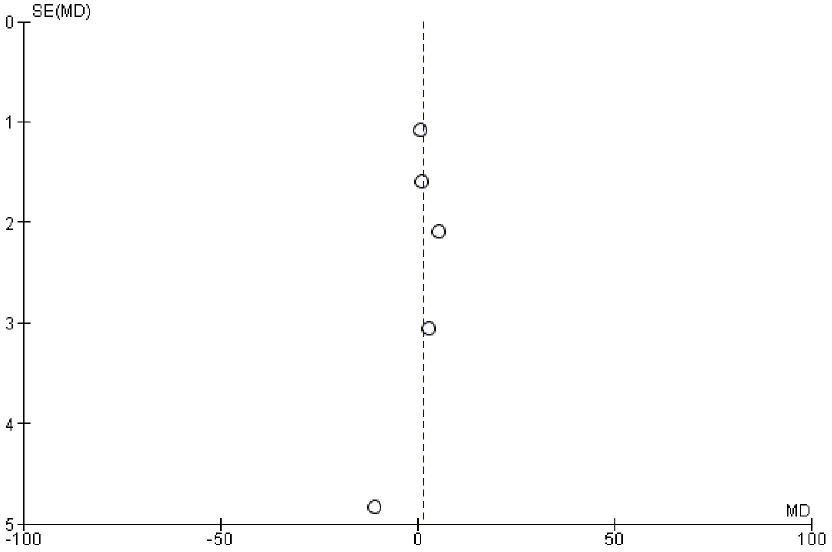

C. Duration of mechanical ventilation

C-1. Risk-of-bias summary: Review authors' judgements about each risk of bias item for each included study.

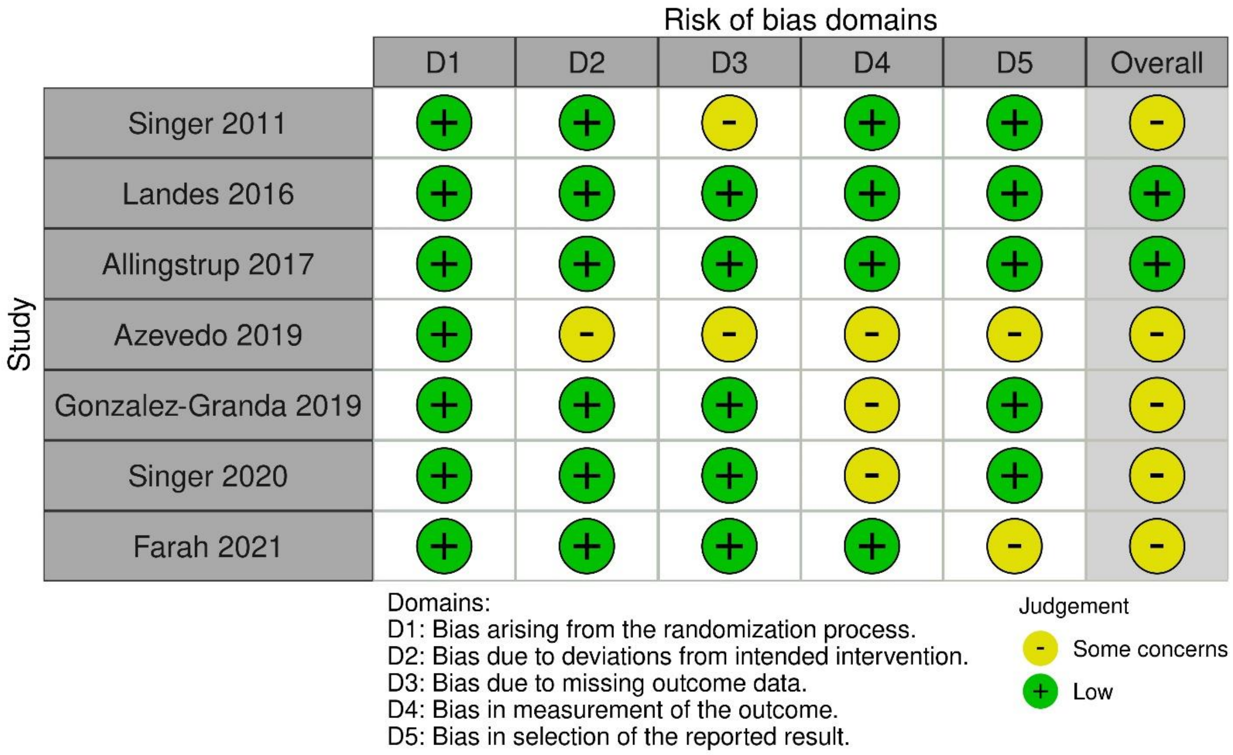

C-2. Risk-of-bias graph: Review authors' judgements about each risk of bias item presented as percentages across all included studies.

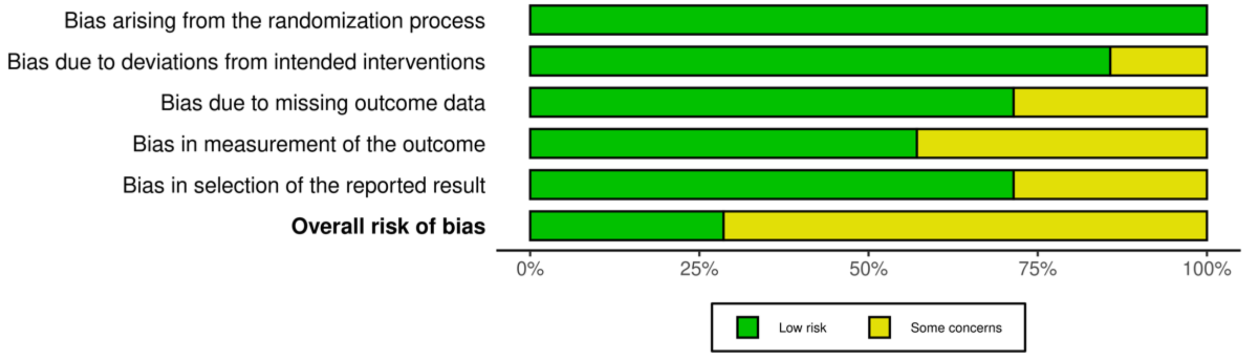

C-3. Forest plot of comparison: Duration of mechanical ventilation

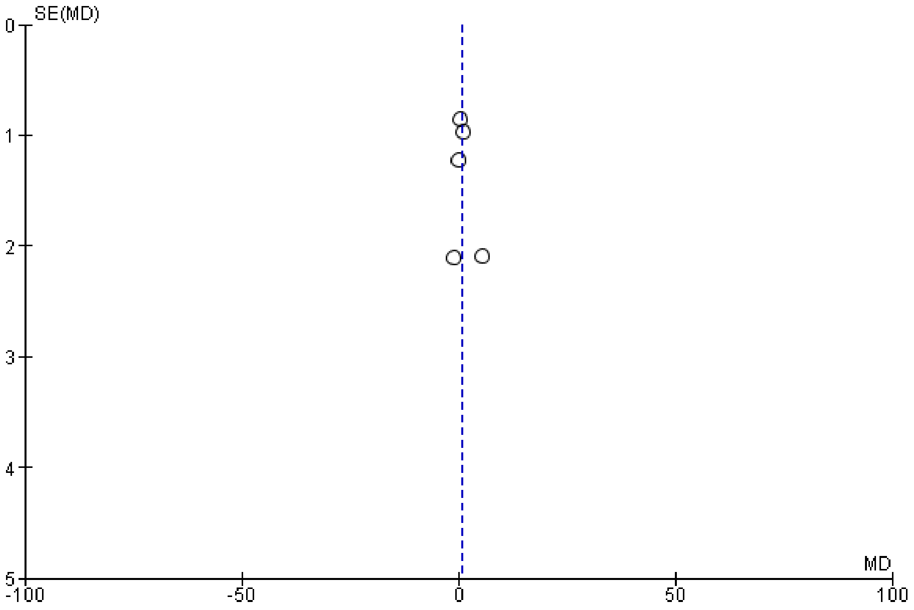

D. All infections

D-1. Risk-of-bias summary: Review authors' judgements about each risk of bias item for each included study.

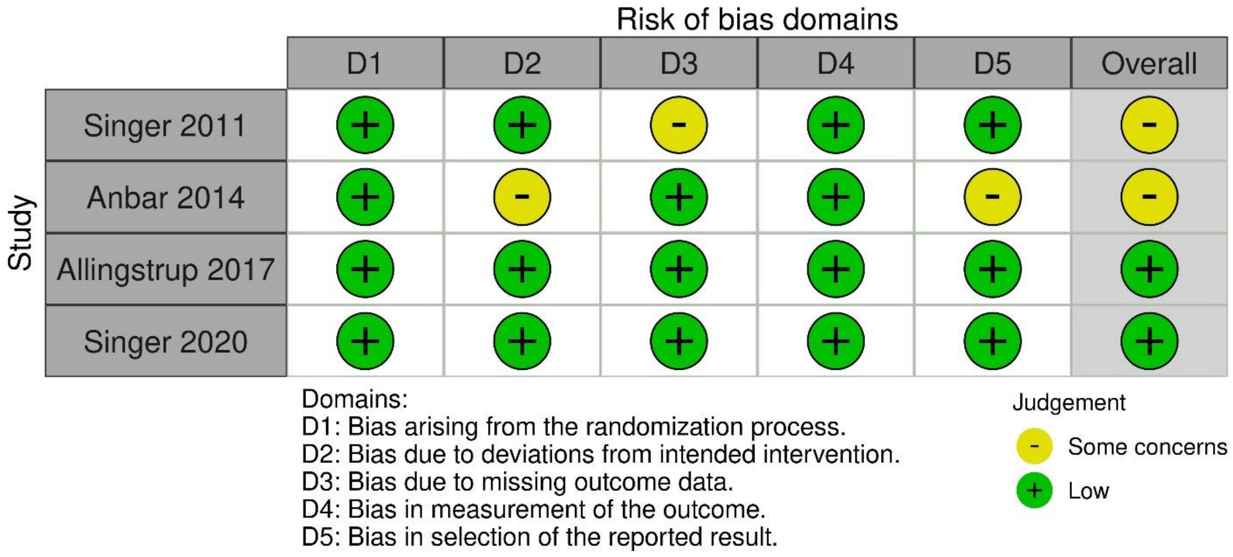

D-2. Risk-of-bias graph: Review authors' judgements about each risk of bias item presented as percentages across all included studies.

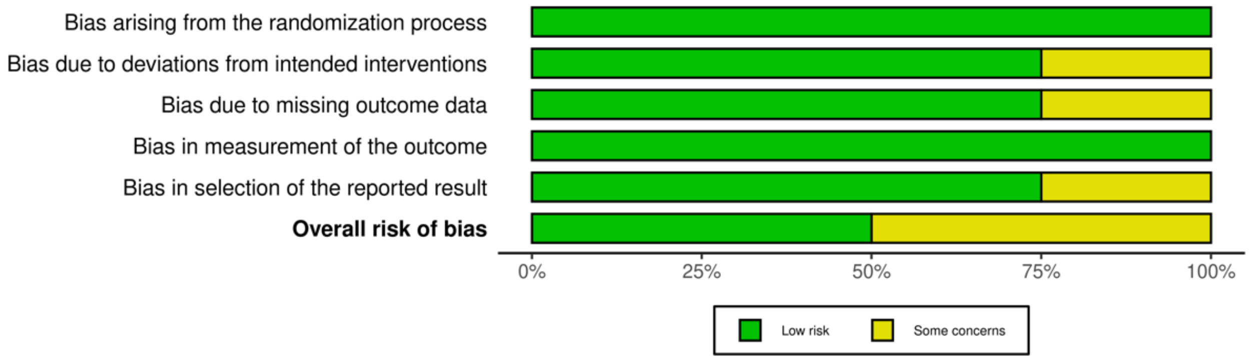

D-3. Forest plot of comparison: All infections

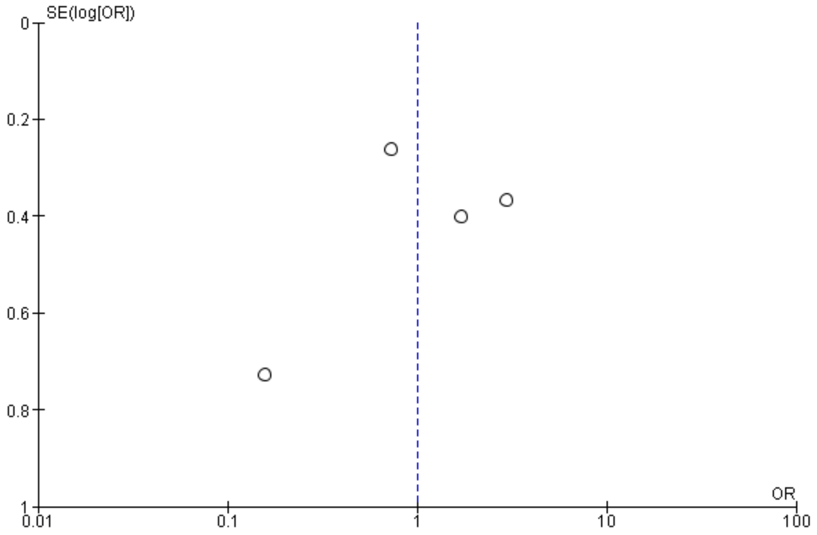

E. Ventilator-associated pneumonia

E-1. Risk-of-bias summary: Review authors' judgements about each risk of bias item for each included study.

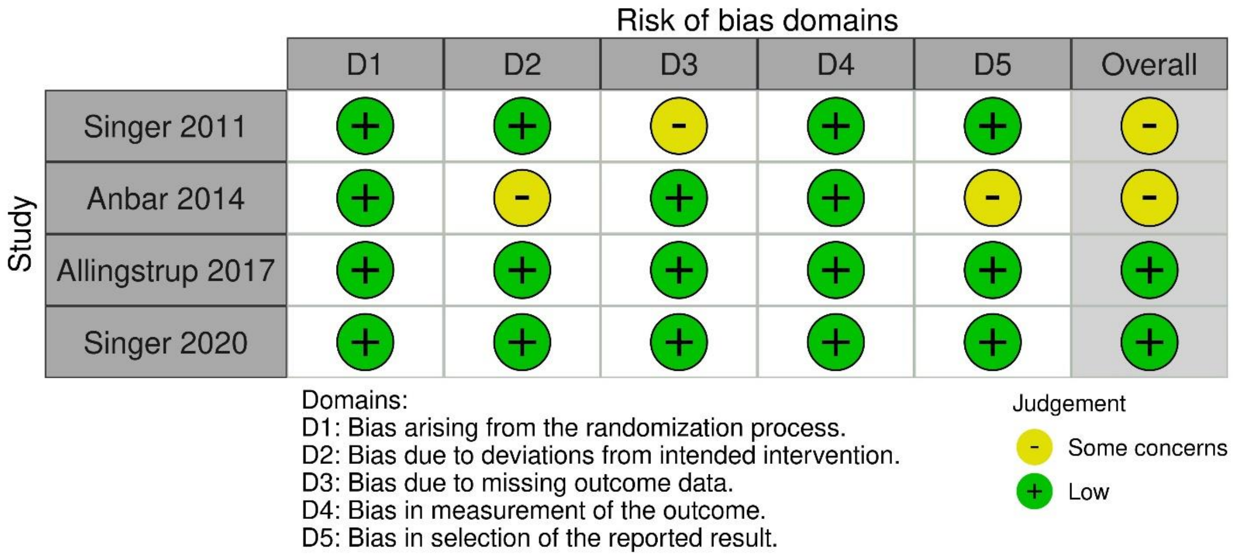

E-2. Risk-of-bias graph: Review authors' judgements about each risk of bias item presented as percentages across all included studies.

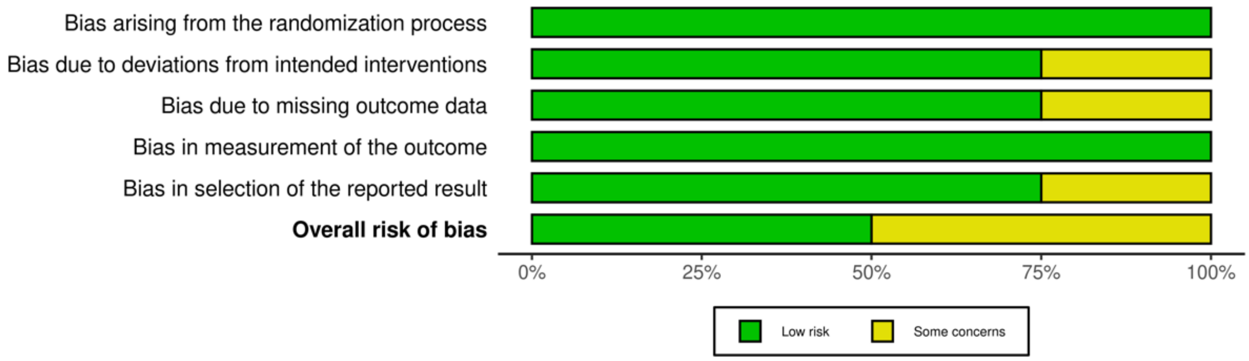

E-3. Forest plot of comparison: Ventilator-associated pneumonia

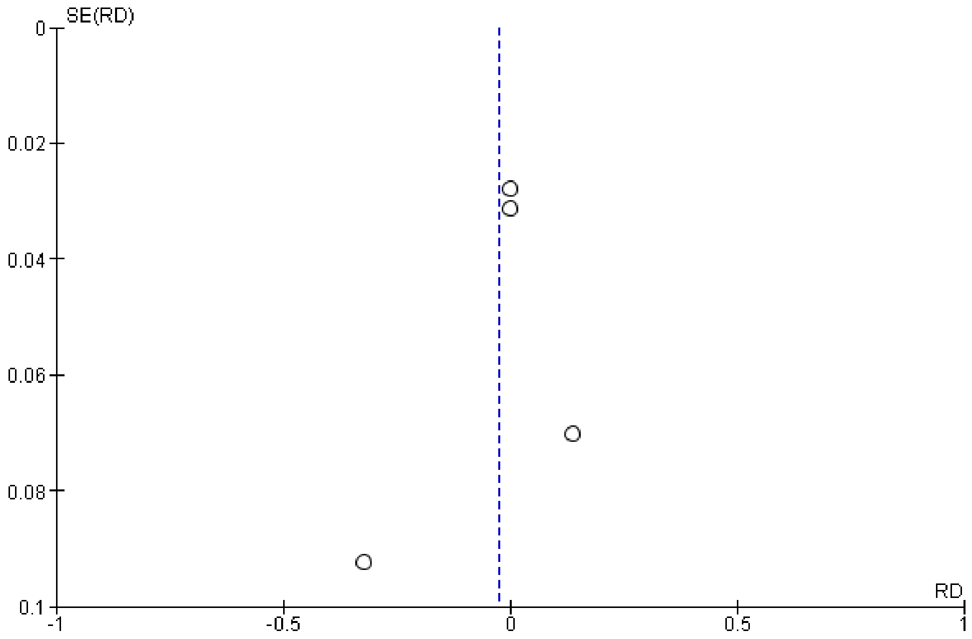

F. Physical functions (physical component summary)

F-1. Risk-of-bias summary: Review authors' judgements about each risk of bias item for each included study.

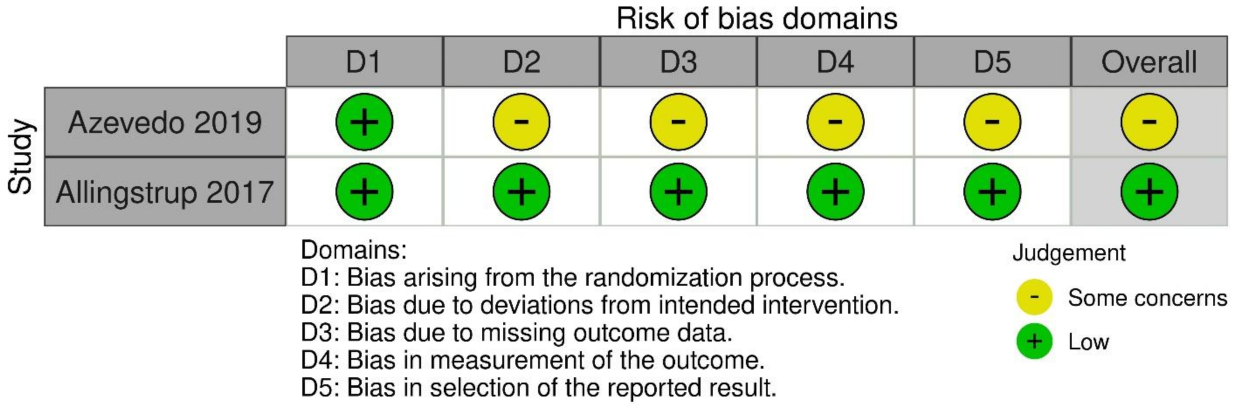

F-2. Risk-of-bias graph: Review authors' judgements about each risk of bias item presented as percentages across all included studies.

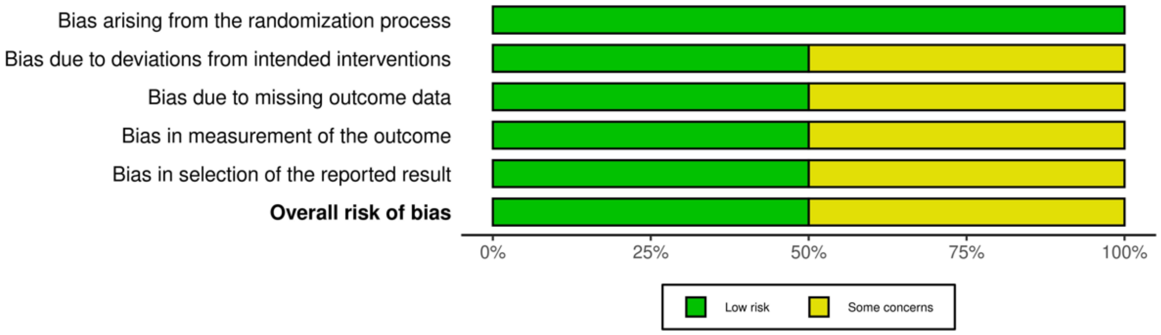

F-3. Forest plot of comparison: Physical functions (physical component summary)

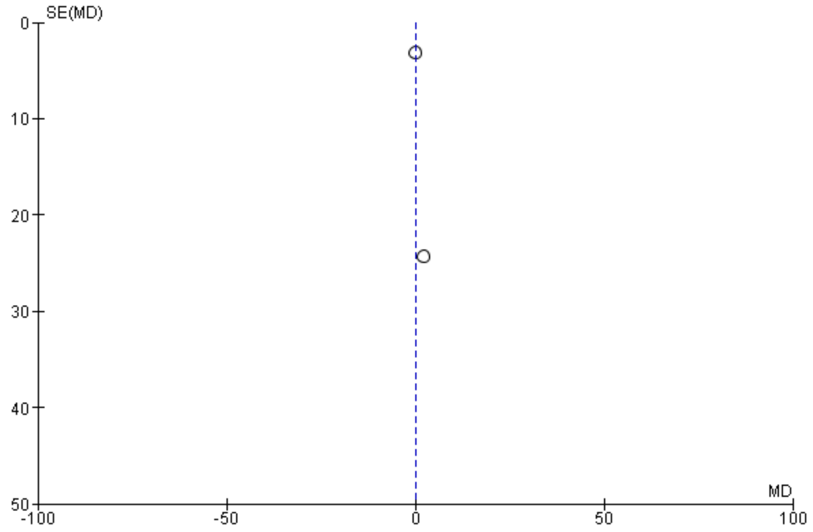

G. Adverse events (kidney)

G-1. Risk-of-bias summary: Review authors' judgements about each risk of bias item for each included study.

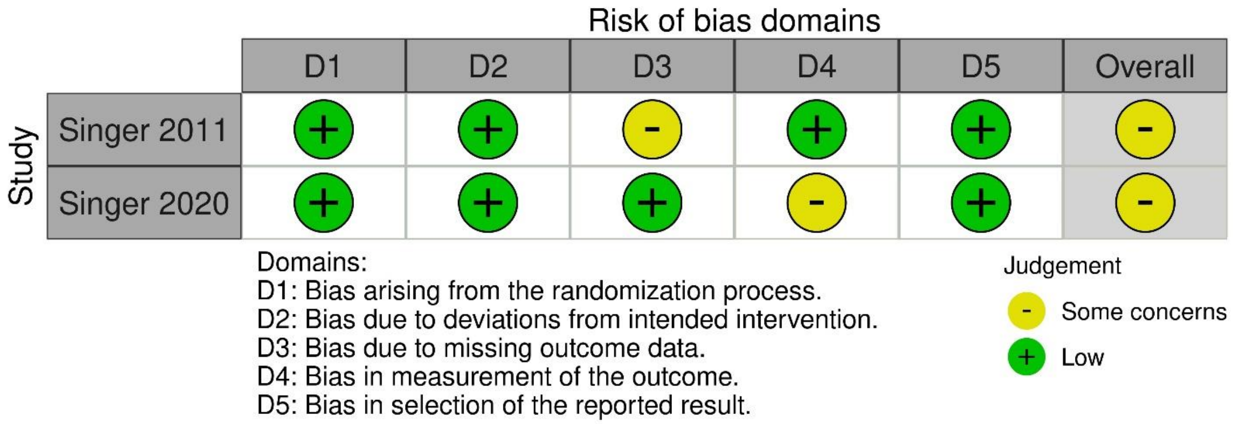

G-2. Risk-of-bias graph: Review authors' judgements about each risk of bias item presented as percentages across all included studies.

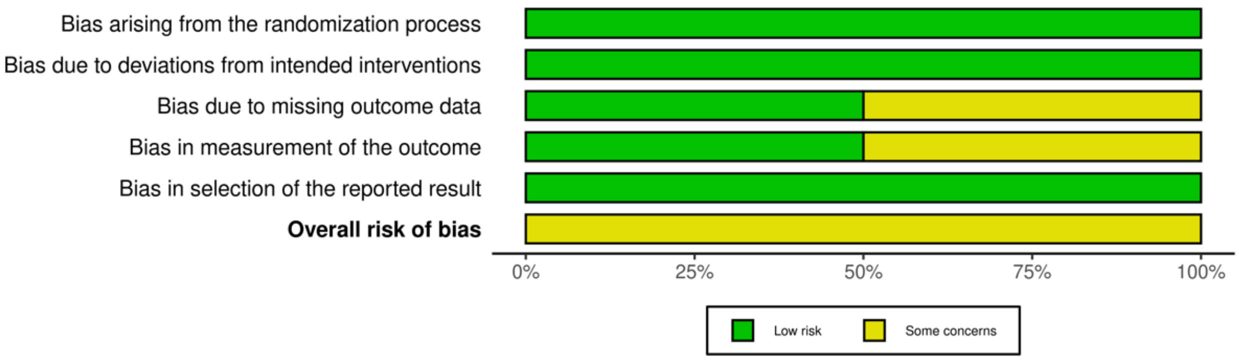

G-3. Forest plot of comparison: Adverse events (kidney)

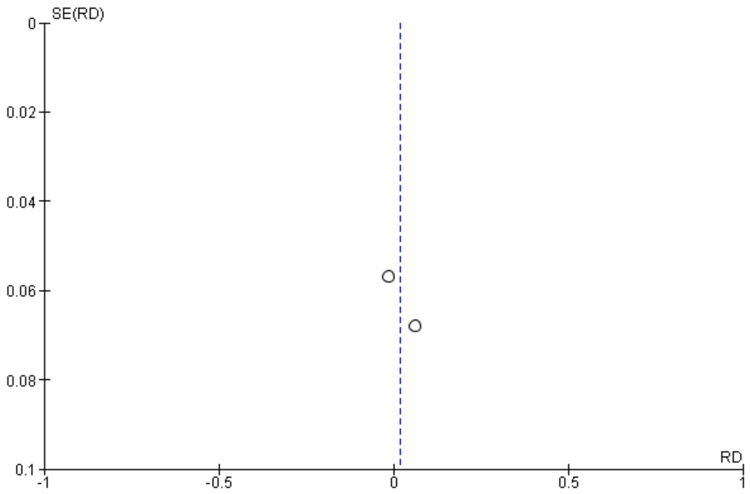

H. Adverse events (liver)

H-1. Risk-of-bias summary: Review authors' judgements about each risk of bias item for each included study.

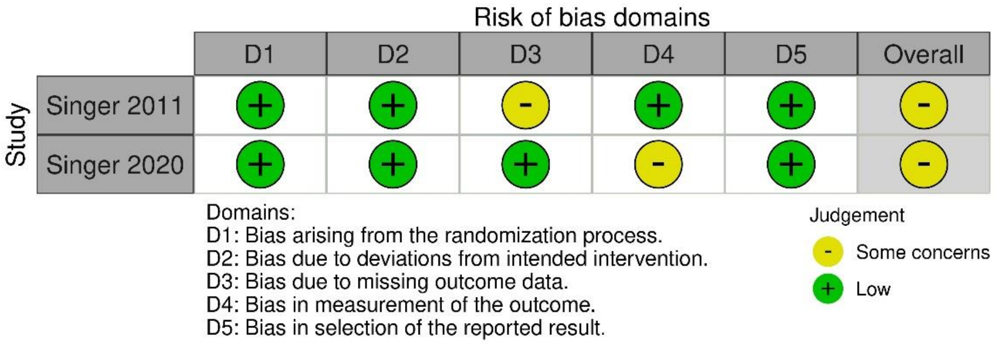

H-2. Risk-of-bias graph: Review authors' judgements about each risk of bias item presented as percentages across all included studies.

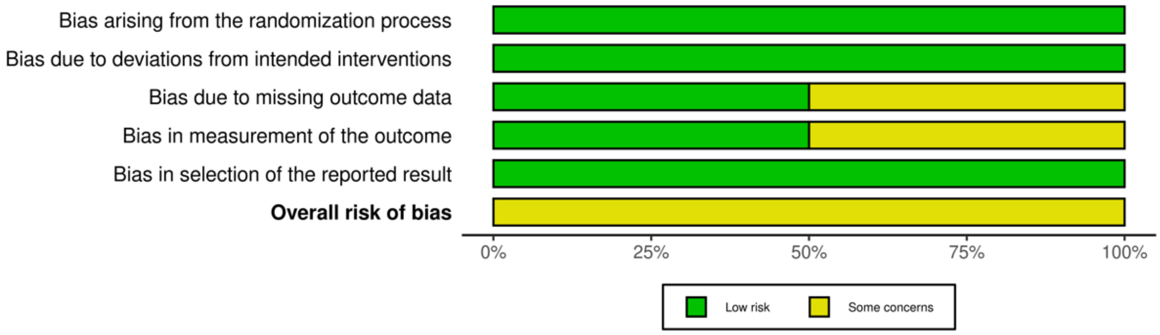

H-3. Forest plot of comparison: Adverse events (liver)

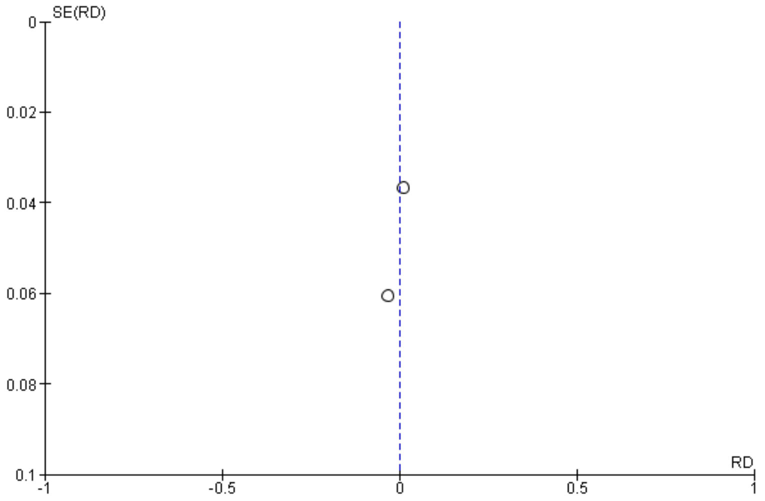

Supplementary Figure S1. Risk-of-bias summary and graph

6. Forest plot for outcomes.

2-1 Short-term mortality

Study or Subgroup	IC Events	IC Total	Equation Events	Equation Total	Weight	Risk Ratio M-H, Random, 95% CI	Year
Jeffrey 1990	3	26	2	23	1.5%	1.33 [0.24, 7.26]	1990
Singer 2011	21	65	31	65	23.5%	0.68 [0.44, 1.05]	2011
Anbar 2014	0	22	2	28	0.5%	0.25 [0.01, 5.00]	2014
Allingtrup 2017	30	100	32	99	26.0%	0.93 [0.61, 1.40]	2017
Gonzales-Granda 2019	5	20	3	20	2.7%	1.67 [0.46, 6.06]	2019
Azevedo 2019	26	57	29	63	29.2%	0.99 [0.67, 1.46]	2019
Singer 2020	22	200	29	200	16.5%	0.76 [0.45, 1.27]	2020
**Total (95% CI)**		**490**		**498**	**100.0%**	**0.86 [0.70, 1.06]**	
Total events	107		128				

Heterogeneity: $Tau^2 = 0.00$; $Chi^2 = 3.94$, $df = 6$ ($P = 0.68$); $I^2 = 0\%$
Test for overall effect: $Z = 1.38$ ($P = 0.17$)

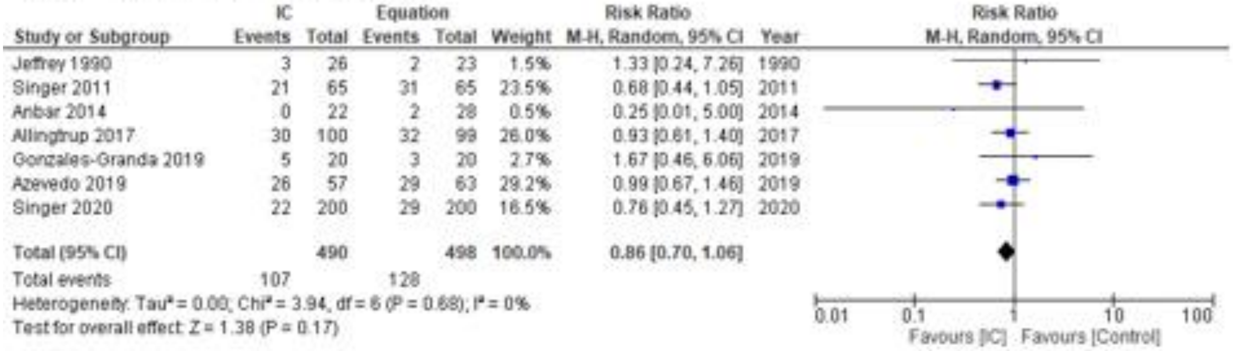

2-2 Length of ICU stay

Study or Subgroup	IC Mean	IC SD	IC Total	Equation Mean	Equation SD	Equation Total	Weight	Mean Difference IV, Random, 95% CI	Year
Jeffrey 1990	48.8	4.5	26	48.5	5.2	23	18.2%	0.30 [-2.44, 3.04]	1990
Singer 2011	17.2	14.6	65	11.7	8.4	65	12.1%	5.50 [1.41, 9.59]	2011
Allingtrup 2017	8	14.8	100	7	5.9	99	16.2%	1.00 [-2.12, 4.12]	2017
Azevedo 2019	21	14.8	57	18	18.5	63	7.2%	3.00 [-2.97, 8.97]	2019
Gonzales-Granda 2019	13	8	20	24	20	20	3.4%	-11.00 [-20.44, -1.56]	2019
Singer 2020	13.1	12.5	199	12.2	8.9	207	21.7%	0.90 [-1.22, 3.02]	2020
Farah 2021	9.4	5.6	73	9.7	7.8	73	21.2%	-0.30 [-2.50, 1.90]	2021
**Total (95% CI)**			**540**			**550**	**100.0%**	**0.86 [-0.98, 2.70]**	

Heterogeneity: $Tau^2 = 2.90$; $Chi^2 = 12.69$, $df = 6$ ($P = 0.05$); $I^2 = 53\%$
Test for overall effect: $Z = 0.92$ ($P = 0.36$)

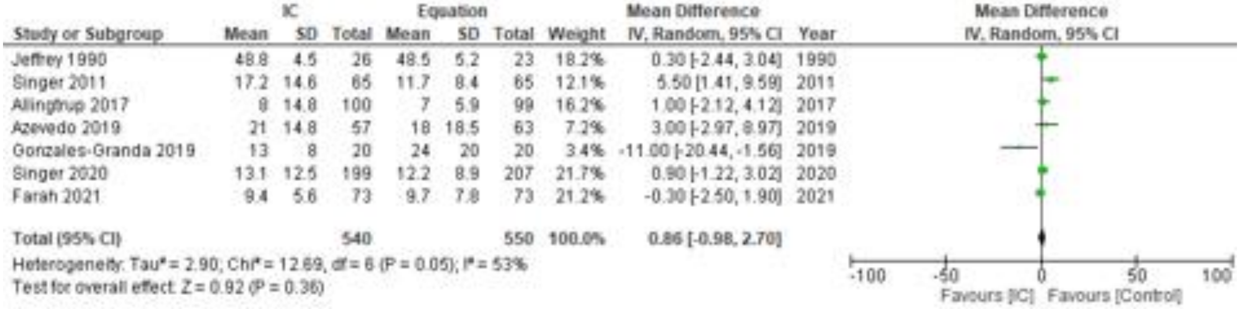

2-3 Duration of mechanical ventilation

Study or Subgroup	IC Mean	IC SD	IC Total	Equation Mean	Equation SD	Equation Total	Weight	Mean Difference IV, Random, 95% CI	Year
Singer 2011	16.1	14.7	65	10.5	8.3	65	6.2%	5.60 [1.50, 9.70]	2011
Landes 2016	48.6	21.7	15	46	31.2	12	0.3%	2.60 [-18.19, 23.39]	2016
Allingtrup 2017	6	8.1	100	5	5.2	99	23.9%	1.00 [-0.89, 2.89]	2017
Azevedo 2019	9	6.67	57	9	6.67	63	16.4%	0.00 [-2.39, 2.39]	2019
Gonzales-Granda 2019	9	8	20	10	5	20	6.1%	-1.00 [-5.13, 3.13]	2019
Singer 2020	10.2	9.3	199	9.8	8	207	28.2%	0.40 [-1.29, 2.09]	2020
Farah 2021	8.3	5.3	73	8.2	7.9	73	19.0%	0.10 [-2.08, 2.28]	2021
**Total (95% CI)**			**529**			**539**	**100.0%**	**0.66 [-0.39, 1.72]**	

Heterogeneity: $Tau^2 = 0.29$; $Chi^2 = 6.98$, $df = 6$ ($P = 0.32$); $I^2 = 14\%$
Test for overall effect: $Z = 1.23$ ($P = 0.22$)

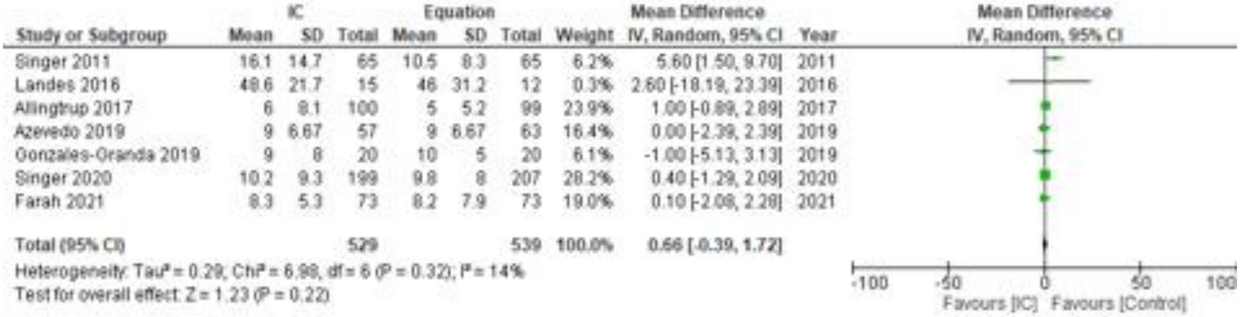

2-4 All infections

Study or Subgroup	IC Events	IC Total	Equation Events	Equation Total	Weight	Risk Ratio M-H, Random, 95% CI	Year
Singer 2011	37	65	20	65	29.0%	1.85 [1.21, 2.82]	2011
Anbar 2014	3	22	14	28	17.2%	0.27 [0.09, 0.83]	2014
Allingtrup 2017	19	100	12	99	24.7%	1.57 [0.80, 3.05]	2017
Singer 2020	31	199	42	207	29.0%	0.77 [0.50, 1.17]	2020
**Total (95% CI)**		**386**		**399**	**100.0%**	**0.99 [0.51, 1.93]**	
Total events	90		88				

Heterogeneity: $Tau^2 = 0.36$; $Chi^2 = 15.95$, $df = 3$ ($P = 0.001$); $I^2 = 81\%$
Test for overall effect: $Z = 0.03$ ($P = 0.98$)

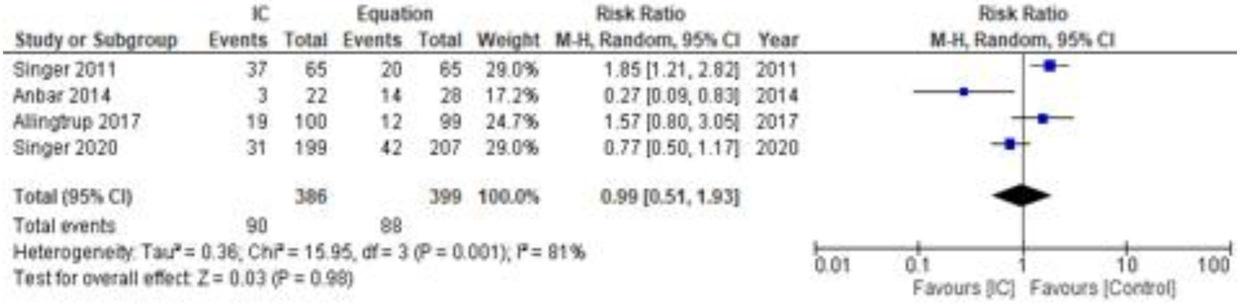

2-5 Ventilator-associated pneumonia

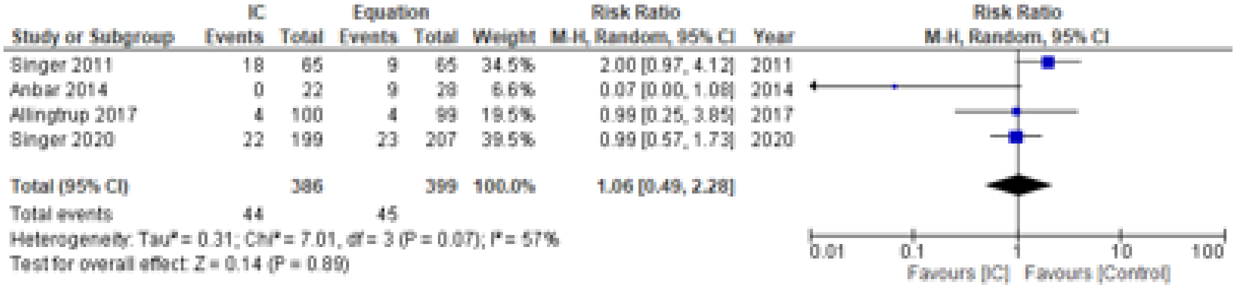

Study or Subgroup	IC Events	IC Total	Equation Events	Equation Total	Weight	Risk Ratio M-H, Random, 95% CI	Year
Singer 2011	18	65	9	65	34.5%	2.00 [0.97, 4.12]	2011
Anbar 2014	0	22	9	28	6.6%	0.07 [0.00, 1.08]	2014
Allingtrup 2017	4	100	4	99	19.5%	0.99 [0.25, 3.85]	2017
Singer 2020	22	199	23	207	39.5%	0.99 [0.57, 1.73]	2020
**Total (95% CI)**		**386**		**399**	**100.0%**	**1.06 [0.49, 2.28]**	
Total events	44		45				

Heterogeneity: Tau² = 0.31; Chi² = 7.01, df = 3 (P = 0.07); I² = 57%
Test for overall effect: Z = 0.14 (P = 0.89)

2-6 Physical functions (physical component summary)

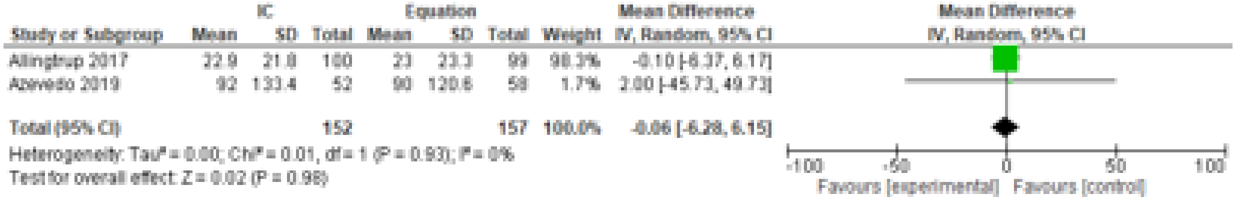

Study or Subgroup	IC Mean	IC SD	IC Total	Equation Mean	Equation SD	Equation Total	Weight	Mean Difference IV, Random, 95% CI
Allingtrup 2017	22.9	21.8	100	23	23.3	99	98.3%	-0.10 [-6.37, 6.17]
Azevedo 2019	92	133.4	52	90	120.6	58	1.7%	2.00 [-45.73, 49.73]
**Total (95% CI)**			**152**			**157**	**100.0%**	**-0.06 [-6.28, 6.15]**

Heterogeneity: Tau² = 0.00; Chi² = 0.01, df = 1 (P = 0.93); I² = 0%
Test for overall effect: Z = 0.02 (P = 0.98)

2-7 Adverse events (kidney)

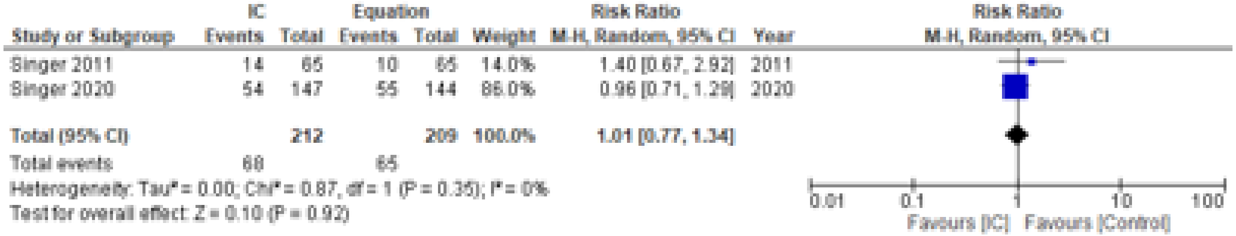

Study or Subgroup	IC Events	IC Total	Equation Events	Equation Total	Weight	Risk Ratio M-H, Random, 95% CI	Year
Singer 2011	14	65	10	65	14.0%	1.40 [0.67, 2.92]	2011
Singer 2020	54	147	55	144	86.0%	0.96 [0.71, 1.29]	2020
**Total (95% CI)**		**212**		**209**	**100.0%**	**1.01 [0.77, 1.34]**	
Total events	68		65				

Heterogeneity: Tau² = 0.00; Chi² = 0.87, df = 1 (P = 0.35); I² = 0%
Test for overall effect: Z = 0.10 (P = 0.92)

2-8 Adverse events (liver)

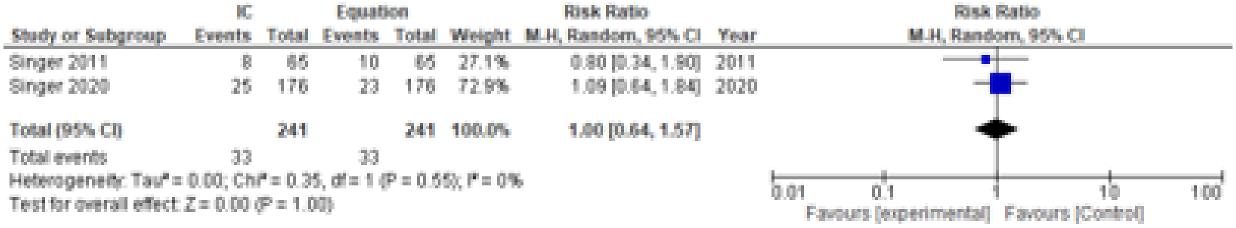

Study or Subgroup	IC Events	IC Total	Equation Events	Equation Total	Weight	Risk Ratio M-H, Random, 95% CI	Year
Singer 2011	8	65	10	65	27.1%	0.80 [0.34, 1.90]	2011
Singer 2020	25	176	23	176	72.9%	1.09 [0.64, 1.84]	2020
**Total (95% CI)**		**241**		**241**	**100.0%**	**1.00 [0.64, 1.57]**	
Total events	33		33				

Heterogeneity: Tau² = 0.00; Chi² = 0.35, df = 1 (P = 0.55); I² = 0%
Test for overall effect: Z = 0.00 (P = 1.00)

Supplementary Figure S2. Forest plot for outcomes.

2-1. Short-term mortality. 2-2. Length of ICU stay. 2-3. Duration of mechanical ventilation. 2-4. All infections. 2-5. Adverse events (kidney). 2-6. Adverse events (liver).

7. Post-hoc analyses of primary outcomes

3.1 Short-term mortality (frequency of IC measurements)

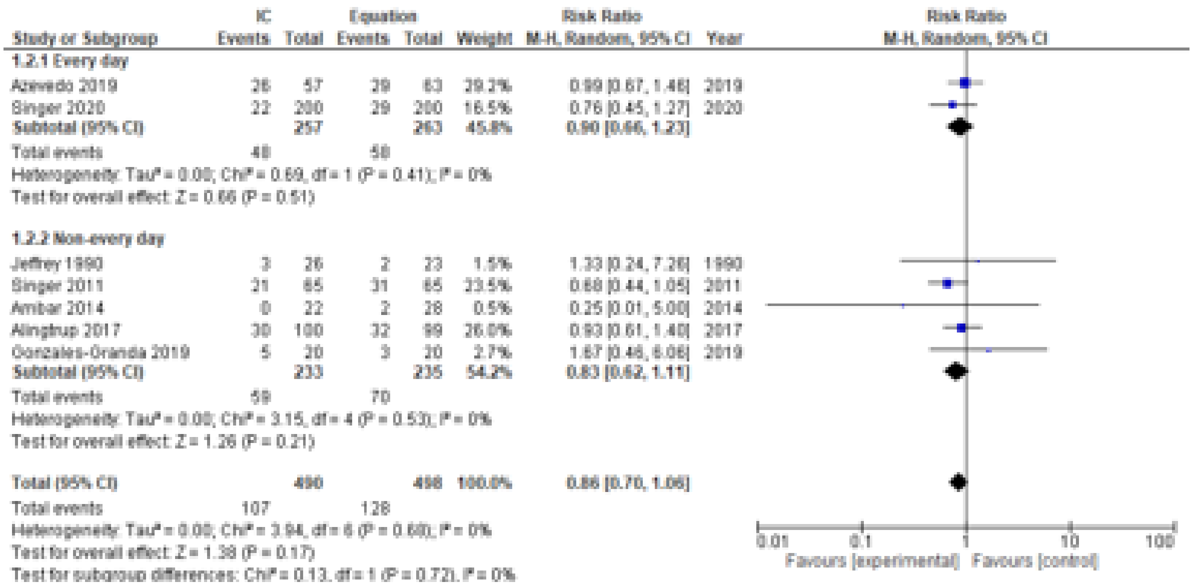

Study or Subgroup	IC Events	IC Total	Equation Events	Equation Total	Weight	Risk Ratio M-H, Random, 95% CI	Year
**1.2.1 Every day**							
Azevedo 2019	26	57	29	63	29.2%	0.99 [0.67, 1.46]	2019
Singer 2020	22	200	29	200	16.5%	0.76 [0.45, 1.27]	2020
**Subtotal (95% CI)**		**257**		**263**	**45.8%**	**0.90 [0.66, 1.23]**	
Total events	48		58				
Heterogeneity: Tau² = 0.00; Chi² = 0.69, df = 1 (P = 0.41); I² = 0%							
Test for overall effect: Z = 0.66 (P = 0.51)							
**1.2.2 Non-every day**							
Jeffrey 1990	3	26	2	23	1.5%	1.33 [0.24, 7.26]	1990
Singer 2011	21	65	31	65	23.5%	0.68 [0.44, 1.05]	2011
Ambar 2014	0	22	2	28	0.5%	0.25 [0.01, 5.00]	2014
Allingtrup 2017	30	100	32	99	26.0%	0.93 [0.61, 1.40]	2017
Gonzales-Oranda 2019	5	20	3	20	2.7%	1.67 [0.46, 6.06]	2019
**Subtotal (95% CI)**		**233**		**235**	**54.2%**	**0.83 [0.62, 1.11]**	
Total events	59		70				
Heterogeneity: Tau² = 0.00; Chi² = 3.15, df = 4 (P = 0.53); I² = 0%							
Test for overall effect: Z = 1.26 (P = 0.21)							
**Total (95% CI)**		**490**		**498**	**100.0%**	**0.86 [0.70, 1.06]**	
Total events	107		128				
Heterogeneity: Tau² = 0.00; Chi² = 3.94, df = 6 (P = 0.68); I² = 0%							
Test for overall effect: Z = 1.38 (P = 0.17)							
Test for subgroup differences: Chi² = 0.13, df = 1 (P = 0.72), I² = 0%							

3.2 Short-term mortality (delivery calories)

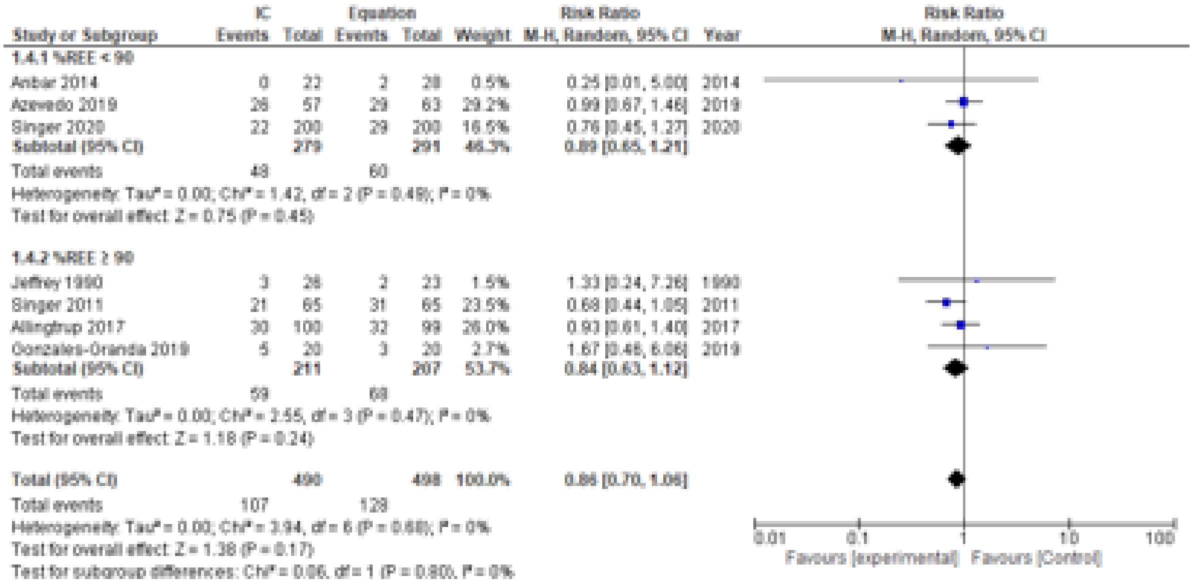

Study or Subgroup	IC Events	IC Total	Equation Events	Equation Total	Weight	Risk Ratio M-H, Random, 95% CI	Year
**1.4.1 %REE < 90**							
Anbar 2014	0	22	2	28	0.5%	0.25 [0.01, 5.00]	2014
Azevedo 2019	26	57	29	63	29.2%	0.99 [0.67, 1.46]	2019
Singer 2020	22	200	29	200	16.5%	0.76 [0.45, 1.27]	2020
**Subtotal (95% CI)**		**279**		**291**	**46.3%**	**0.89 [0.65, 1.21]**	
Total events	48		60				
Heterogeneity: Tau² = 0.00; Chi² = 1.42, df = 2 (P = 0.49); I² = 0%							
Test for overall effect: Z = 0.75 (P = 0.45)							
**1.4.2 %REE ≥ 90**							
Jeffrey 1990	3	26	2	23	1.5%	1.33 [0.24, 7.26]	1990
Singer 2011	21	65	31	65	23.5%	0.68 [0.44, 1.05]	2011
Allingtrup 2017	30	100	32	99	26.0%	0.93 [0.61, 1.40]	2017
Gonzales-Oranda 2019	5	20	3	20	2.7%	1.67 [0.46, 6.06]	2019
**Subtotal (95% CI)**		**211**		**207**	**53.7%**	**0.84 [0.63, 1.12]**	
Total events	59		68				
Heterogeneity: Tau² = 0.00; Chi² = 2.55, df = 3 (P = 0.47); I² = 0%							
Test for overall effect: Z = 1.18 (P = 0.24)							
**Total (95% CI)**		**490**		**498**	**100.0%**	**0.86 [0.70, 1.06]**	
Total events	107		128				
Heterogeneity: Tau² = 0.00; Chi² = 3.94, df = 6 (P = 0.68); I² = 0%							
Test for overall effect: Z = 1.38 (P = 0.17)							
Test for subgroup differences: Chi² = 0.06, df = 1 (P = 0.80), I² = 0%							

3.3 Short-term mortality (mechanically ventilated patients)

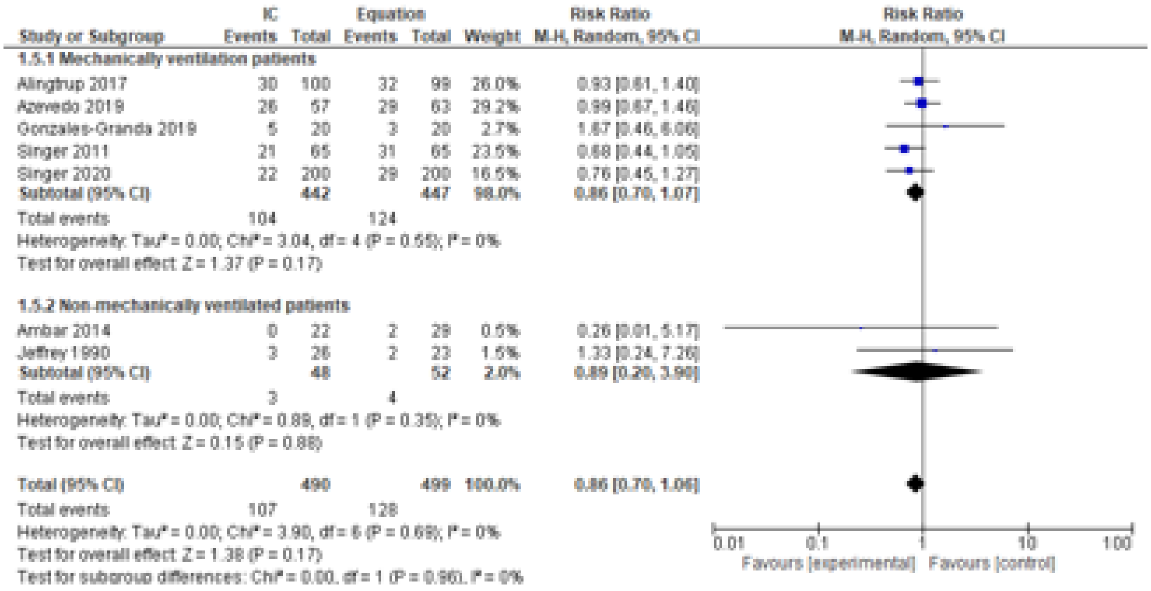

Supplementary Figure S3. Post-hoc analyses of primary outcomes

8. Subgroup analysis

2-1. Short-term mortality (burn patients)

Study or Subgroup	IC Events	IC Total	Equation Events	Equation Total	Weight	Risk Ratio M-H, Random, 95% CI	Year
**1.1.1 Burn patients**							
Jeffrey 1990	3	26	2	23	1.5%	1.33 [0.24, 7.26]	1990
**Subtotal (95% CI)**		**26**		**23**	**1.5%**	**1.33 [0.24, 7.26]**	
Total events	3		2				
Heterogeneity: Not applicable							
Test for overall effect: Z = 0.33 (P = 0.74)							
**1.1.2 Non burn patients**							
Singer 2011	21	65	31	65	23.5%	0.68 [0.44, 1.05]	2011
Anbar 2014	0	22	2	28	0.5%	0.25 [0.01, 5.00]	2014
Allingtrup 2017	30	100	32	99	26.0%	0.93 [0.61, 1.40]	2017
Gonzales-Granda 2019	5	20	3	20	2.7%	1.67 [0.46, 6.06]	2019
Azevedo 2019	26	57	29	63	29.2%	0.99 [0.67, 1.46]	2019
Singer 2020	22	200	29	200	16.5%	0.76 [0.45, 1.27]	2020
**Subtotal (95% CI)**		**464**		**475**	**98.5%**	**0.86 [0.69, 1.06]**	
Total events	104		126				
Heterogeneity: Tau² = 0.00; Chi² = 3.69, df = 5 (P = 0.59); I² = 0%							
Test for overall effect: Z = 1.43 (P = 0.15)							
**Total (95% CI)**		**490**		**498**	**100.0%**	**0.86 [0.70, 1.06]**	
Total events	107		128				
Heterogeneity: Tau² = 0.00; Chi² = 3.94, df = 6 (P = 0.68); I² = 0%							
Test for overall effect: Z = 1.38 (P = 0.17)							
Test for subgroup differences: Chi² = 0.25, df = 1 (P = 0.62), I² = 0%							

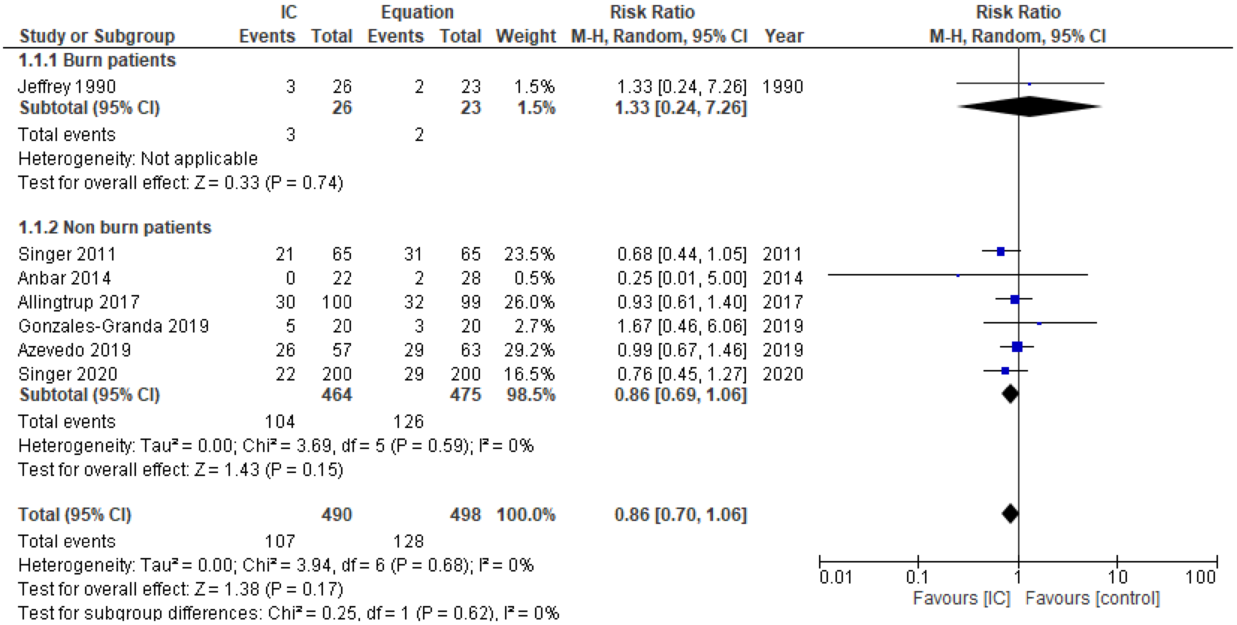

Supplementary Figure S4. Subgroup analysis of primary outcomes

9. Evidence profile

Certainty assessment							Summary of findings				
Participants (studies) Follow-up	Risk of bias	Inconsistency	Indirectness	Imprecision	Publication bias	Overall certainty of evidence	Study event rates (%)		Relative effect (95% CI)	Anticipated absolute effects	
							With Equation	With IC		Risk with Equation	Risk difference with IC
**Short-term mortality**											
988 (7 RCTs)	not serious	not serious	not serious	serious[a]	none	⊕⊕⊕○ Moderate	128/498 (25.7%)	107/490 (21.8%)	**RR 0.86** (0.70 to 1.06)	257 per 1,000	**36 fewer per 1,000** (from 77 fewer to 15 more)
**Length of ICU stay**											
1090 (7 RCTs)	not serious	serious[b]	not serious	serious[c]	none	⊕⊕○○ Low	550	540	-		MD **0.86 higher** (0.98 lower to 2.7 higher)
**Duration of mechanical ventilation**											
1068 (7 RCTs)	not serious	not serious	not serious	serious[c]	none	⊕⊕⊕○ Moderate	539	529	-		MD **0.66 higher** (0.39 lower to 1.72 higher)
**All infections**											
785 (4 RCTs)	not serious	very serious[d]	not serious	serious[a]	none	⊕○○○ Very low	88/399 (22.1%)	90/386 (23.3%)	**RR 0.99** (0.51 to 1.93)	221 per 1,000	**13 more per 1,000** (from 40 fewer to 82 more)
**Ventilator-associated pneumonia**											
785 (4 RCTs)	not serious	serious[b]	not serious	serious[a]	none	⊕⊕○○ Low	45/399 (11.3%)	44/386 (11.4%)	**RR 1.06** (0.49 to 2.28)	113 per 1,000	**2 more per 1,000** (from 35 fewer to 58 more)
**Physical functions (physical component summary)**											
309 (2 RCTs)	not serious	not serious	not serious	serious[c]	none	⊕⊕⊕○ Moderate	152	157	-		**MD 0.06 lower** (6.28 lower to 6.15 higher)
**Adverse events (kidney)**											
421 (2 RCTs)	not serious	not serious	not serious	serious[a]	none	⊕⊕⊕○ Moderate	65/209 (31.1%)	68/212 (32.1%)	**RR 1.01** (0.77 to 1.34)	311 per 1,000	**9 more per 1,000** (from 68 fewer to 112 more)
**Adverse events (liver)**											
482 (2 RCTs)	not serious	not serious	not serious	serious[a]	none	⊕⊕⊕○ Moderate	33/241 (13.7%)	33/241 (13.7%)	**RR 1.00** (0.64 to 1.57)	137 per 1,000	**0 fewer per 1,000** (from 49 fewer to 78 more)

Supplemental table S5. Evidence profile

CI: confidence interval; **MD:** mean difference; **RR:** risk ratio; **IC**: indirect calorimetric; **ICU**: intensive care unit.

Explanations

a. Downgraded one point for imprecision: because the sample size is less than N=2000 (calculate OIS based on α=0.05, β=0.2, Event=20%, RRR=25%, N=2000)

b. Downgraded one point for inconsistency: because the percentage of variation between studies (I2) is high

c. Downgraded one point for imprecision: because the sample size is less than N=800 (calculate OIS based on empirical thresholds; α=0.05, β=0.2, d=0.2~0.3, N=800)

d. Downgraded two points for inconsistency: because the percentage of variation between studies (I2) is high and significant in the heterogeneity test
